# Integrative analysis of toxicometabolomics and toxicoproteomics data: new molecular insights into thiazolidinedione-induced cardiotoxicity

**DOI:** 10.1007/s11306-024-02201-3

**Published:** 2024-12-04

**Authors:** Abdullah Al Sultan, Zahra Rattray, Nicholas J. W. Rattray

**Affiliations:** 1 Strathclyde Institute of Pharmacy and Biomedical Sciences, University of Strathclyde, 161 Cathedral Street, Glasgow, G4 0RE UK; 2 Faculty of Pharmacy, Kuwait University, Safat, 13110 Kuwait

**Keywords:** Thiazolidinediones, Toxicometabolomics, Toxicoproteomics, Cardiotoxicity, Mitochondrial energetics, Oxidative stress

## Abstract

**Introduction:**

Despite the well-established efficacy of thiazolidinediones (TZDs), including pioglitazone and rosiglitazone, in type II diabetes management, their potential contribution to heart failure risk remains a significant area of uncertainty. This incomplete understanding, which persists despite decades of clinical use of TZDs, has generated ongoing controversy and unanswered questions regarding their safety profiles, ultimately limiting their broader clinical application.

**Objective and methods:**

This study presented a multi-omics approach, integrating toxicoproteomics and toxicometabolomics data with the goal of uncovering novel mechanistic insights into TZD cardiotoxicity and identifying molecular signatures predictive of side effect progression.

**Results:**

Network analysis of proteo-metabolomic data revealed a distinct fingerprint of disrupted biochemical pathways, which were primarily related to energy metabolism. Downregulation of oxidative phosphorylation and fatty acid synthesis was coupled with increased activity in anaerobic glycolysis, the pentose phosphate pathway, and amino acid and purine metabolism. This suggests a potential metabolic shift in AC16 cells from fatty acid oxidation towards anaerobic glycolysis, potentially contributing to observed cardiotoxicity. Additionally, the study identified a marked disruption in the glutathione system, indicating an imbalanced redox state triggered by TZD exposure. Importantly, our analysis identified key molecular signatures across omics datasets, including prominent signatures of amino acids like L-ornithine, L-tyrosine and glutamine, which are evidently associated with heart failure, supporting their potential use for the early prediction of cardiotoxicity progression.

**Conclusion:**

By uncovering a novel mechanistic explanation for TZD cardiotoxicity, this study simultaneously illuminates potential therapeutic interventions, opening avenues for future research to improve the safety profile of TZD agents. (**250 words**)

**Graphical abstracts:**

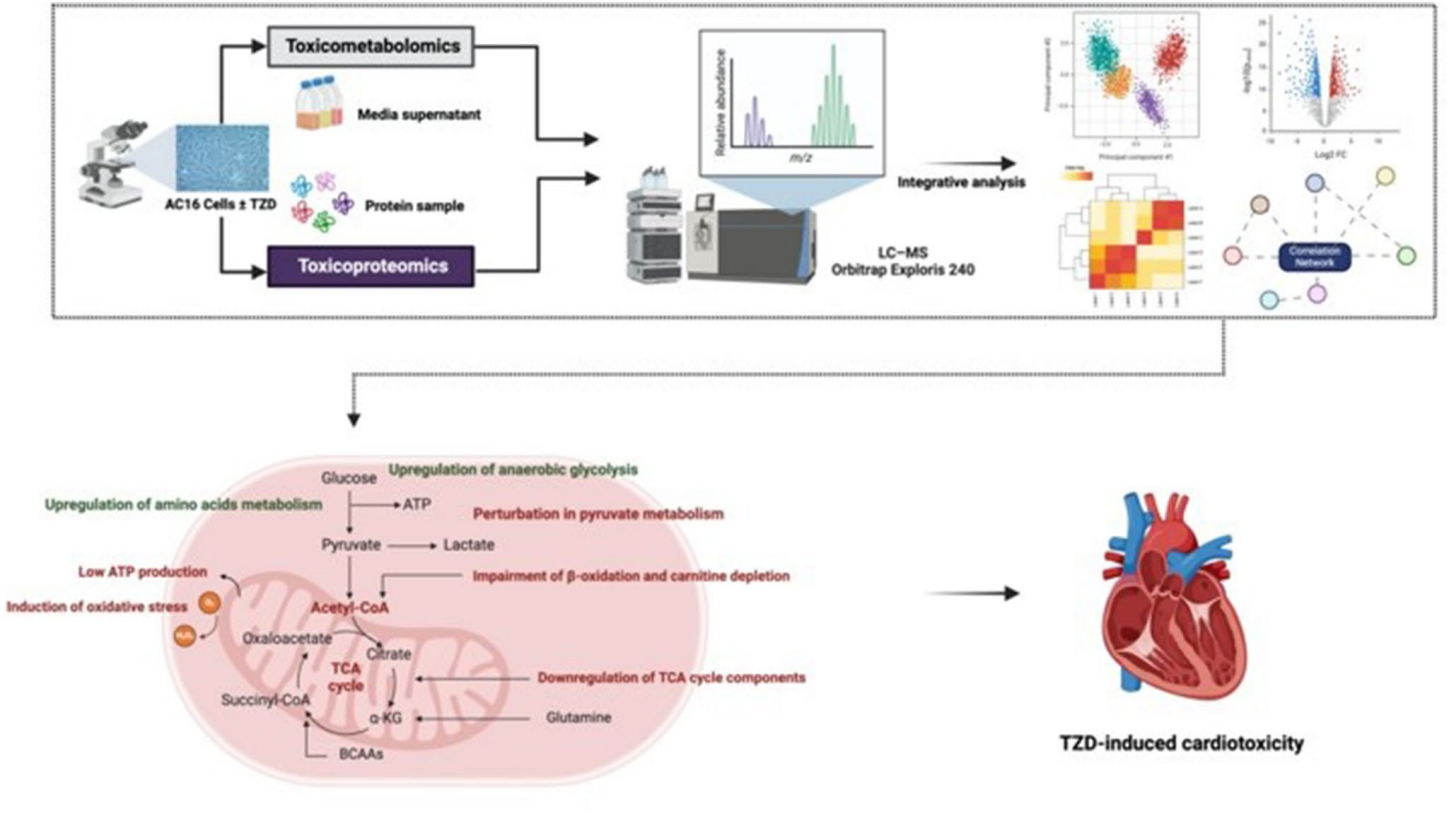

**Supplementary Information:**

The online version contains supplementary material available at 10.1007/s11306-024-02201-3.

## Introduction

Within the last decade, the concept of medication safety has risen to the forefront of the healthcare and drug development agenda in recognition of the fact that it plays a pivotal role in patients’ clinical outcomes (Alshammari, 2016). The overarching purpose of medication safety is to prevent or at least reduce the occurrence of adverse drug reactions (ADRs), which are broadly defined by the National Patient Safety Agency (NPSA) in the UK as ‘any unintended or unexpected incident which could have or did lead to harm for one or more patients’ (Courtenay & Griffiths, 2010). According to the latest US Centers for Disease Control and Prevention (CDC) report, ADRs necessitate 1.3 million emergency department visits annually (Thacker et al., 2020). It has also been estimated by the CDC that 350,000 patients per year require hospitalisation following emergency visits for ADRs (Thacker et al., 2020). Within the context of ADRs and drug development, a recent study has proposed four main reasons for the 90% failure rate of drug development, one being related to drug toxicity, which accounts for 30% of the attrition of drug candidates (Sun et al., 2022). Given the deleterious impact of ADRs, collectively, on healthcare and drug development processes, comprehensive toxicological-based studies are urgently needed to screen and further elucidate the toxicity mechanisms implicated in medication ADRs.

Conventional in vivo and cellular systems toxicological-based approaches have been principally adopted to investigate the ADRs-induced drug development failure of medications, mainly through observing targeted toxicological endpoints. Although these conventional methods have yielded crucial outcomes, they have several drawbacks. They are time-consuming, and their primary focus, namely, on identifying and testing limited molecular targets, is often unlikely to fully characterise the safety profile of a drug. A growing body of single- and multi-omics-based approaches has therefore emerged as powerful tools in toxicological research, providing comprehensive and unprecedented mechanistic insights capable of filling the existing data gaps and hence improving our understanding of drug toxicity (Hu & Jia, 2021; Li et al., 2021; Marx-Stoelting et al., 2015; Nguyen et al., 2022). The implications of both single- and multi-omics-based toxicological studies have effectively shifted the landscape of toxicological investigation from an observational-based strategy to a more mechanical and target-based analysis of the impact of chemicals on the human system (Marx-Stoelting et al., 2015). Various omics approaches currently exist, which include toxicogenomics, toxicotranscriptomics, toxicometabolomics, and toxicoproteomics, each of which has provided unparalleled insights into the toxicity pathways of various chemicals at different molecular levels (i.e., DNA, RNA, and protein to metabolites) in a high-throughput manner and an acceptable time-frame (Marx-Stoelting et al., 2015; Nguyen et al., 2022). Notably, it is evident that most of the toxicological studies performed thus far have adopted single-omics-based approaches (Hu & Jia, 2021; Li et al., 2022; Nury et al., 2023; Olesti et al., 2021; Zaitsu et al., 2016). Despite the extensive findings of these studies, the single-omics-derived data are mainly associative and lack the resolving power required to establish causality between observed molecular perturbations and phenotypic manifestations. These concerns about the single-omics approach have led to a revolution in omics study design and a paradigm shift toward integrating a multi-omics-based approach (Hu & Jia, 2021; Li et al., 2021). Recent papers have implied that the application of the multi-omics approach has provided novel and compelling opportunities to establish causality across different cellular function levels; thus, it has become the cutting edge of ADR research (Chen et al., 2020; Hu & Jia, 2021; Nguyen et al., 2022; Xie et al., 2020).

Recognising the transformative power of multi-omics integration in toxicology research, and with the aim of expanding upon our prior solo-omics investigation (Al Sultan et al., 2024a, b), this study was designed to integrate our toxicometabolomics and toxicoproteomics analyses of human adult cardiomyocytes AC16 treated with a class of anti-diabetic agents named thiazolidinedione (TZD). TZDs, represented by pioglitazone (PGZ) and rosiglitazone (ROSI) agents, are cost-effective anti-diabetic agents used in the management of type-II diabetes mellitus (T2DM) (Wajid et al., 2019). Despite their efficacious profile, clinical cases of heart failure (HF) have been reported, hampering their clinical application (Administration, 2010, 2012). Since the mechanisms of TZD-induced cardiotoxicity are yet to be fully unravelled, the current study introduced a novel liquid chromatography–mass spectrometry (LC–MS)-based multi-omics pipeline designed to (i) integrate potential relationships among the key identified metabolites and proteins and (ii) identify novel protein-metabolite modules capable of elucidating previously undiscovered biochemical pathways perturbed in TZD toxicity.

## Methods

### Reagents and chemicals

TZDs, PGZ and ROSI, were obtained from Sigma-Aldrich. The LC-MS analysis used reagents purchased from Fisher Scientific: high-performance liquid chromatography (HPLC)-grade acetonitrile, methanol, analytical-grade formic acid, and ultrapure water.

### Cells and cell culture

The AC16 cell line was purchased from Sigma-Aldrich and cultured in Dulbecco’s Modified Eagle’s Medium (DMEM/F-12) supplemented with 12.5% foetal bovine serum (FBS), 1% antibiotics (streptomycin and penicillin) and 2 mM L-glutamine at 37 °C in a humid atmosphere of 5% CO_2_ and 95% air.

### LC–MS-based toxicometabolomics analysis

A comprehensive description of the methodologies used for sample preparation, metabolite extraction, and LC-MS data acquisition can be found in our previous work (Al Sultan et al., 2024b) and Supplementary Sect. 1.1. For data processing, Compound Discoverer was used, and features identified in the processed raw data of mass spectral peaks, within a 5-ppm mass error, were searched against the mzCloud spectral library and ChemSpider™ databases. The databases selected by ChemSpider included the Human Metabolome Database (HMDB), BioCyc, Chemical Entities of Biological Interest (ChEBI), Kyoto Encyclopedia of Genes and Genomes (KEGG), Taneisa Grier, Toxin, Toxin-Target Database, WikiPathways, and xPharm. All reported data align with MSI Level 2 identification. No in-house chemical standards were run alongside the metabolomics samples during the analysis. Further details about the parameters used during processing are provided in our previous work (Al Sultan et al., 2024b) and Supplementary Sect. 1.1.3.

### LC–MS-based toxicoproteomics analysis

Details of the specific methodologies employed for each stage of the proteomic analysis, including protein extraction, trypsin digestion, microflow LC-MS data acquisition and processing, can be found in (Al Sultan et al., 2024a) and Supplementary Sect. 1.2.

### Integration paradigms and bioinformatic analyses

#### Data-Driven Analysis

To integrate the shotgun toxicoproteomics and toxicometabolomics data, an *N-integration* framework, namely the Data Integration Analysis for Biomarker Discovery using Latent cOmponents (DIABLO) embedded in the *mixOmics* R package, was employed (Rohart et al., 2017). The applied DIABLO model (code documented in Supplementary Sect. 1.1.4), also referred as multiblock sparse partial least squares discriminant analysis (MB-sPLS-DA), imposes sparseness within the latent components and hence was utilised to dissect discriminative omics features across omics datasets while concurrently performing simultaneous dimension reduction (Rohart et al., 2017). Regarding MB-sPLS-DA parameter selection and performance evaluation, a design matrix tuned to 0.1 was used to accentuate the discrimination between genotype groups during analysis. The classification performance was evaluated using the repeated cross-validation through the *perf* function (Rohart et al., 2017). Five-fold cross-validation repeated 50 times was used, and the classification error rate (overall and balanced error rate [BER]) resulting from the cross-validation process across different numbers of components was then recorded for each type of prediction distance (max.dist, centroids.dist, and mahalanobis.dist). The model with the lowest error rate was subsequently chosen to define the optimal number of components for the MB-sPLS-DA model. Lastly, with respect to feature selection, the *tune.block.splsda* function (Rohart et al., 2017) was run with five-fold cross-validation repeated 50 times to determine the suitable number of molecular signatures on each component.

Through analysis of the PGZ datasets using the outlined parameters, the MB-sPLS-DA model constructed with two components yielded the most favourable outcome, namely, the lowest overall estimation error rate (Figure S1a). This, coupled with its excellent discriminatory power, designated it as the optimal model for further investigation. Regarding the supervised integrative analysis of ROSI multi-omics datasets, the optimal number of components on the basis of the performance plot shown in Figure S1b was four; hence, this value was chosen for all downstream analyses.

The MB-sPLS-DA results were primarily visualised using the *mixOmics* R package (Rohart et al., 2017) and the Cytoscape software platform (Cytoscape; https://cytoscape.org; v3.10.1).

#### Knowledge-driven analysis

To gain a holistic understanding of perturbed pathways and their complex interactions across omics levels, a joint pathway analysis of the toxicoproteomics and toxicometabolomics data was performed via the joint-pathway analysis functionality of MetaboAnalyst v6.0 (https://www.metaboanalyst.ca) webserver. First, significance testing was carried out to capture the significant features within each omics dataset. Accordingly, joint pathway analysis of the differential expressed proteins (DEPs) and metabolites/features (DEFs) was configured with the following parameters: a hypergeometric test for enrichment analysis, degree centrality as the topology measure, and a query combination approach for data integration. The KEGG enrichment terms that had a *p-*value < 0.05 were considered to be statistically significant.

### Statistical analysis

Statistical analyses were performed using R software version 4.3.0. All toxicometabolomics and toxicoproteomics data comprised three independent experiments, each run in triplicate (biological replicates), leading to nine samples per group. Student’s t-tests or Welch’s t-tests, dependent on data distribution and variance, were applied to assess statistical significance in pairwise comparisons between the two groups. To compare multiple variables within a single group, a one-way non-repeated ANOVA was followed by Dunnett’s post hoc test for multiple comparisons. The correlation coefficient was assessed using Pearson’s and distance correlation analyses. A *p*-value ≤ 0.05 was defined as the threshold for statistical significance.

**Fig. 1:**
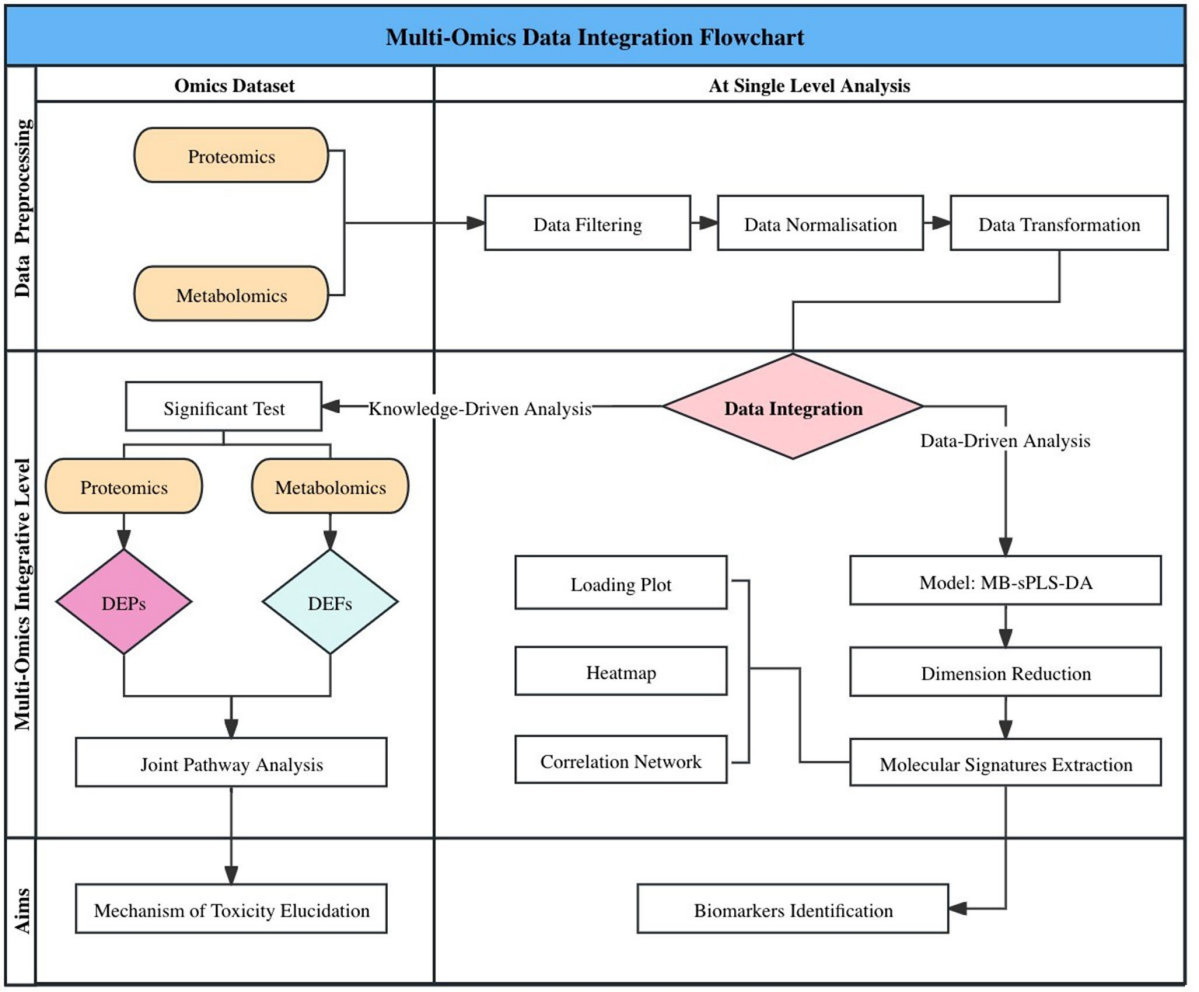
A schematic flow chart of the multi-omics integrative paradigm applied for downstream analyses. Prior to data merging, our previous publications (Al Sultan et al., 2024a, b) reported single-omics analyses comprising toxicometabolomics and toxicoproteomics. In each study, the datasets were subjected to data filtering, normalisation, and transformation, as detailed in the respective publications. For the current analysis, multi-omics data integration was performed using these pre-processed datasets, encompassing both data-driven and knowledge-based approaches (joint pathway analysis). The data-driven analysis employed an MB-sPLS-DA model to assess both inter-omics and intra-omics heterogeneity across sample groups. The model was then trained to identify key molecular signatures within the omics datasets. These identified molecular signatures were visualized in various plots, aimed at identifying potential biomarkers for TZD-induced cardiotoxicity. Furthermore, a significance test was employed within each omics dataset to capture significant features (*p-*value < 0.05), followed by a joint pathway analysis using MetaboAnalyst v6.0 (https://www.metaboanalyst.ca) to holistically comprehend the perturbed pathways underpinning TZD’s adverse effects

MB-sPLS-DA: Multiblock sparse partial least squares discriminant analysis; DEPs: Differential expressed proteins; DEFs: Differential expressed features; TZD: Thiazolidinedione.

A visual summary of the analytical framework, incorporating both data-driven and knowledge-driven components, is presented as a schematic flowchart in Fig. 1.

## Integrative analysis findings of untargeted toxicometabolomics and Toxicoproteomics Data

To comprehensively landscape the heterogeneity among sample groups and unveil a detailed molecular profile of TZD-induced changes in AC16-cardiomyocytes, multivariate analysis integrating proteomics and metabolomics data was employed using the DIABLO framework.

### Multivariate model-driven analysis of AC16 cell proteo-metabolomic response to PGZ exposure

**Fig. 2:**
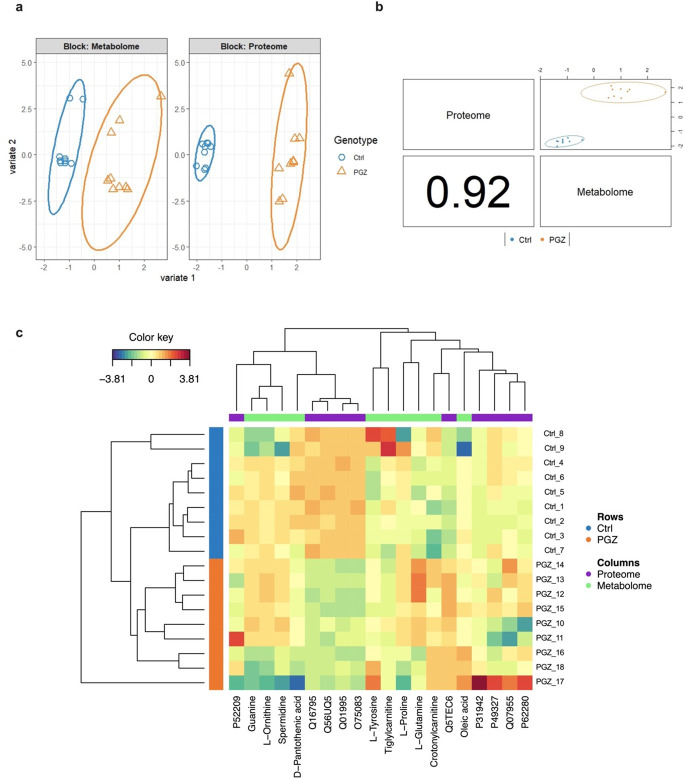
Multiblock supervised partial least squares discriminant analysis (MB-sPLS-DA) model of multi-omics data following PGZ treatment. (**a**) Individual omics dataset contributions to the MB-sPLS-DA model. Score plots revealed distinct separation of control and treated samples at both the metabolome and proteome levels. (**b**) Inter-omics correlations from plotDIABLO displaying the first component in each dataset (upper diagonal plot) and the Pearson correlation between each component (lower diagonal plot), showcasing high correlation between proteomics and metabolomics data. (**c**) Clustered Image Map (Euclidean distance, complete linkage) for the molecular signatures extracted by MB-sPLS-DA performed on the PGZ study. MB-sPLS-DA: Multiblock sparse partial least squares discriminant analysis; PGZ: Pioglitazone

With regard to the PGZ datasets, visual inspection of the sample plots generated by the DIABLO model revealed a distinct separation between the treated and control groups across all omics datasets (Fig. 2a). Furthermore, interrogation of the two omics datasets, as presented in Fig. 2b, yielded a highly significant correlation between their corresponding latent components, indicating a striking level of inter-dataset concordance across heterogeneous data types.

To ensure optimal feature selection for maximal discrimination, a threshold of ± 0.15 was imposed on the loading coefficients of the first and second sPLS-DA components for each data block. This applied criterion for MB-sPLS-DA feature selection (fold = 5, nrepeat = 50, using Mahalanobis as a distance measure) resulted in the identification of five proteins (Table S1) and five metabolites in each component. The multi-omics signature extracted from component 1 included mitochondrial carnitines (tiglylcarnitine, crotonylcarnitine), L-glutamine, oleic acid, and D-pantothenic acid. Prominent protein signatures within this component comprised (6-phosphogluconate dehydrogenase, decarboxylating; P52209), (serine/arginine-rich splicing factor 1; Q07955), (heterogeneous nuclear ribonucleoprotein H3; P31942), (small ribosomal subunit protein uS17; P62280), and (fatty acid synthase; P49327). In contrast to component 1, component 2 exhibited a unique signature comprising metabolites such as spermidine, L-tyrosine, L-proline, and L-ornithine, alongside protein identifications (TPT1-like protein; Q56UQ5), (NADH dehydrogenase [ubiquinone] 1 alpha subcomplex subunit 9, mitochondrial; Q16795), (histone H3-7; Q5TEC6), (WD repeat-containing protein 1; O75083), and (transgelin; Q01995). The contribution of each selected variable to each component across all blocks is illustrated in Figure S2. The expression of each multi-omics molecular signature for each included sample is illustrated in Fig. 2c.

**Fig. 3:**
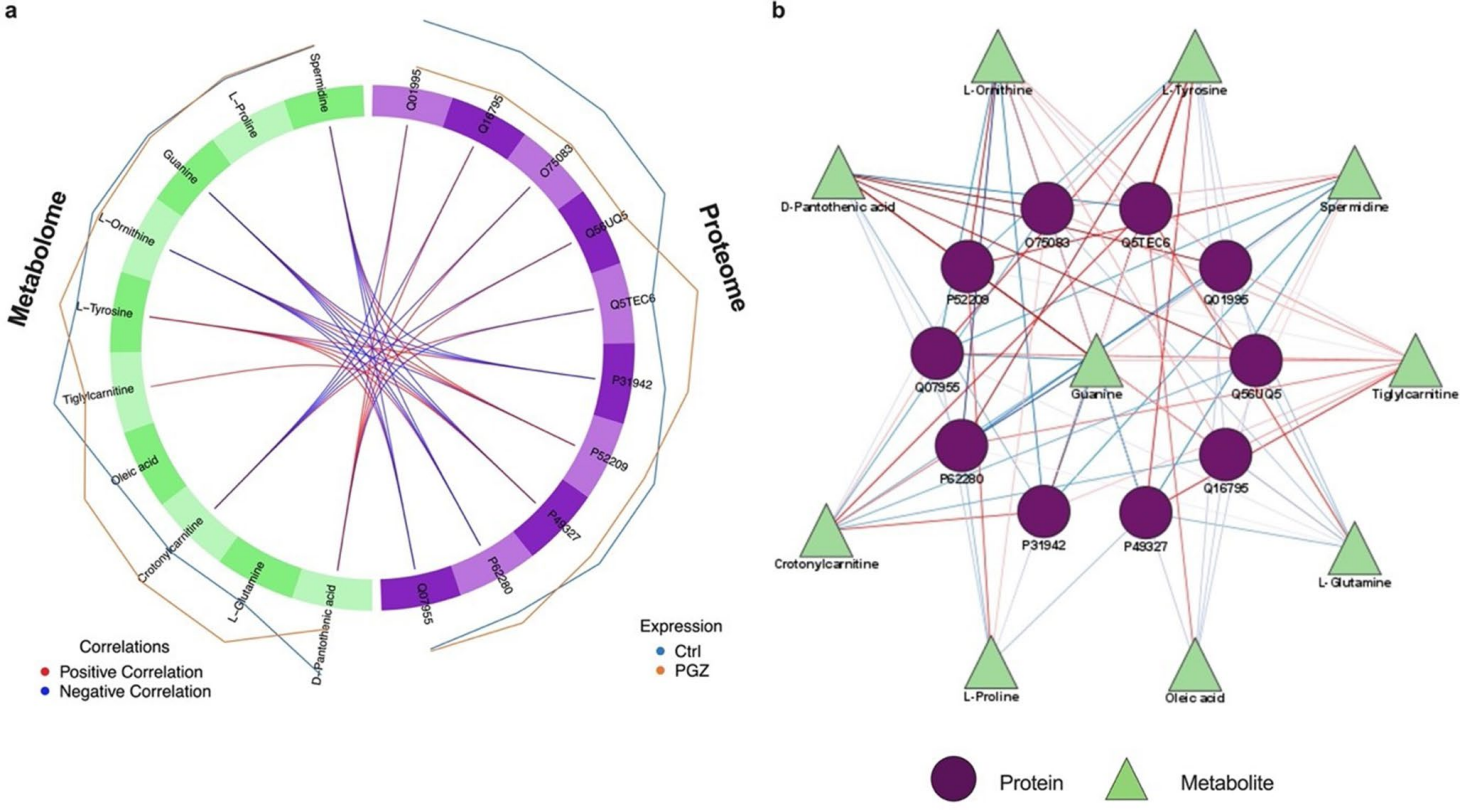
Correlation network analysis of the multi-omics signatures derived by the DIABLO framework. Plot (**a**) displays a circos plot depicting correlations between selected features (cut-off: 0.6), illustrating positive associations in red and negative associations in blue. Plot (**b**) showcases a protein-metabolite interaction network, where circular and triangle shapes represent protein and metabolite features, respectively, and edge colours red and blue represent positive and negative correlations, respectively. The width of the edges represents the strength of correlation. All molecular signatures were included in the network without specifying a cut-off. The protein-metabolite network was generated using (Cytoscape; https://cytoscape.org; v3.10.1)

To visually depict the inter-molecular signature correlations, a circus plot (Fig. 3a) was constructed. The plot revealed dense positive interactions between the following: L-tyrosine and (heterogeneous nuclear ribonucleoprotein H3; P31942)/(small ribosomal subunit protein uS17; P62280); L-ornithine/guanine and (6-phosphogluconate dehydrogenase, decarboxylating; P52209); and D-pantothenic acid with the proteins (TPT1-like protein; Q56UQ5), (NADH dehydrogenase [ubiquinone] 1 alpha subcomplex subunit 9, mitochondrial; Q16795), (transgelin; Q01995), and (WD repeat-containing protein 1; O75083). Further investigation identified robust repulsive/negative interactions involving D-pantothenic acid and (histone H3-7; Q5TEC6); guanine/L-ornithine and the (small ribosomal subunit protein uS17; P62280)/ (fatty acid synthase; P49327)/ (heterogeneous nuclear ribonucleoprotein H3; P31942)/ (serine/arginine-rich splicing factor 1; Q07955) proteins; and spermidine with (small ribosomal subunit protein uS17; P62280), (fatty acid synthase; P49327), and (heterogeneous nuclear ribonucleoprotein H3; P31942). A detailed summary of the potential biological implications of the identified protein-metabolite interactions is presented in Supplementary Table S3.

Lastly, a network of the proteomics and metabolomics key features was constructed based on the similarity matrix (Fig. 3b). Analysis of this proteo-metabolomic network identified a tightly knit cluster of co-regulated features, with tiglylcarnitine, spermidine, L-tyrosine, L-ornithine, and guanine (metabolome block), and (WD repeat-containing protein 1; O75083) and (histone H3-7; Q5TEC6) (proteome block) serving as the prominent hub features that drive this module.

### Multivariate model-driven analysis of ac16 cell proteo-metabolomic response to ROSI exposure

**Fig. 4:**
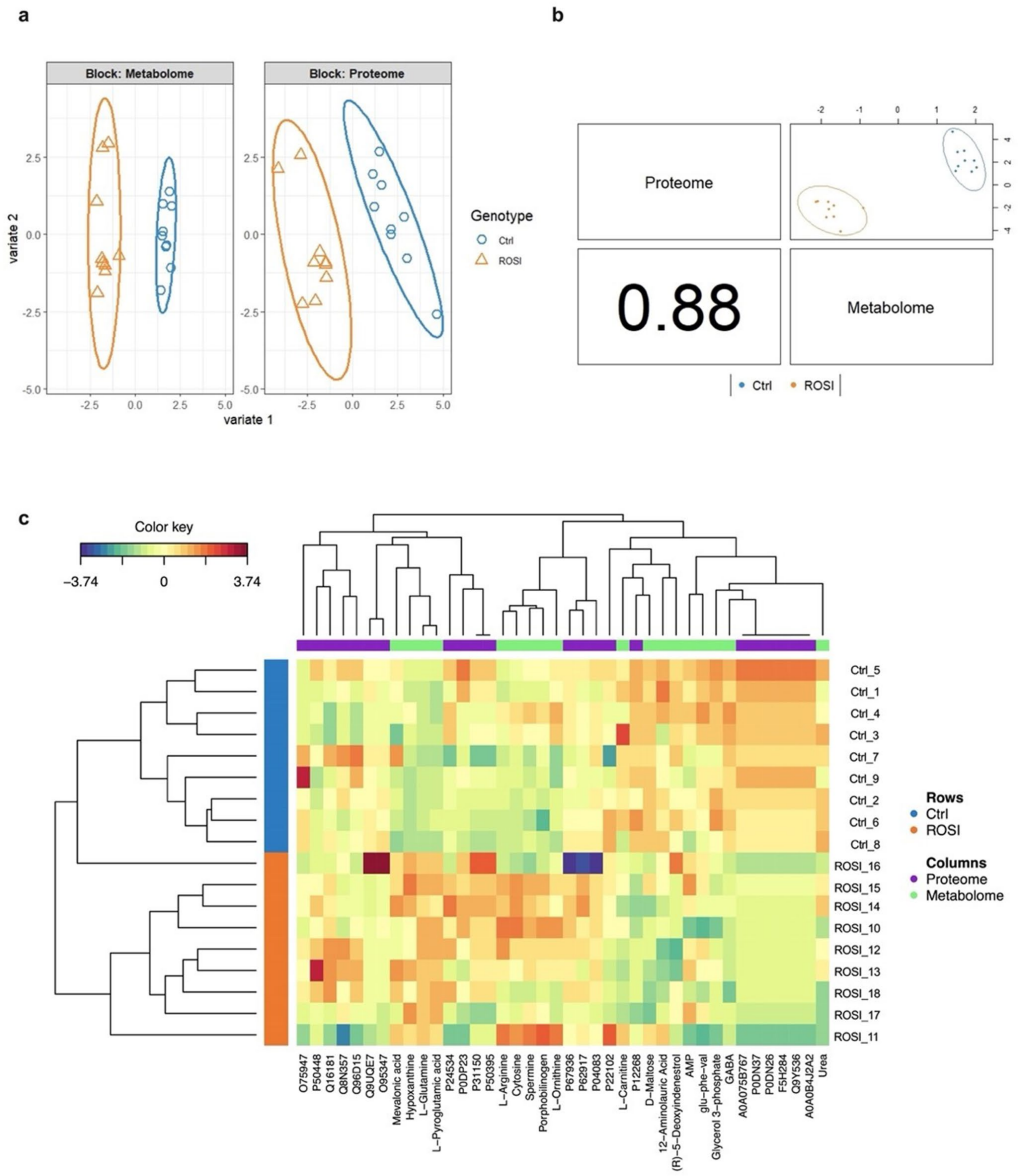
Multi-Omics integration of ROSI datasets via the DIABLO mixOmics framework, (**a**) The individual contribution of each dataset to the MB-sPLS-DA final model, demonstrating distinct intra-omics separation between ROSI-treated samples and control groups. (**b**) Component correlation of each of the two datasets determined by DIABLO analysis, demonstrating a high correlation between the proteomics and metabolomics data. (**c**) Clustered Image Map (Euclidean distance, complete linkage) for the molecular signatures extracted by MB-sPLS-DA performed on the ROSI study MB-sPLS-DA: Multiblock sparse partial least squares discriminant analysis; ROSI: Rosiglitazone

With respect to the ROSI datasets, the DIABLO method, through integrative analysis of inter-omics correlations, pinpointed several crucial features that differentiate ROSI-treated samples from the control group. Visualisation of the sample distribution after projection onto the subspace spanned by components 1 and 2 in Fig. 4a reveals a distinct separation between treated and control groups. Furthermore, and similarly to the PGZ findings, the interrogation of both proteomics and metabolomics datasets, as visually depicted in Fig. 4b, unveiled a highly significant correlation between their respective latent components, signifying a remarkable degree of coherence and agreement between these divergent data modalities.

In terms of feature selection, integrative analysis with DIABLO (fold = 5, nrepeat = 50, using Mahalanobis as a distance measure) identified a signature of 22 proteins (Table S2) and 18 metabolites across all the selected components. The identity of each molecular signature along with its contribution to its perspective component, as well as its expression over the included samples, are illustrated in Figures S3 and 4c.

**Fig. 5:**
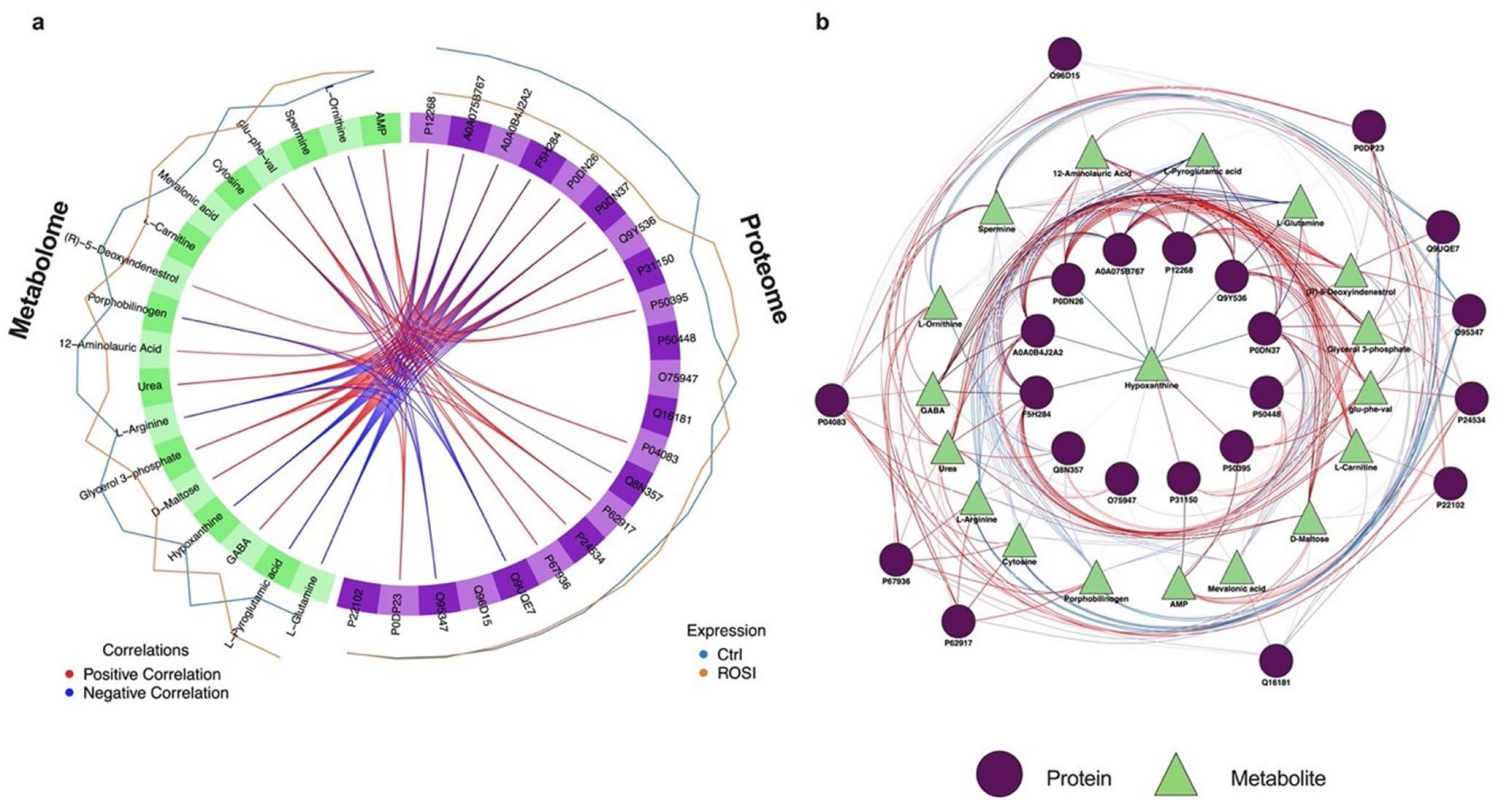
Correlation network analysis of the multi-omics signatures derived by the DIABLO framework. Plot (**a**) displays a circos plot depicting correlations between selected features (cut-off: 0.6), illustrating positive associations in red and negative associations in blue. Plot (**b**) showcases a protein-metabolite interaction network, where circular and triangle shapes represent protein and metabolite features, respectively, and edge colours red and blue represent positive and negative correlations, respectively. The width of the edges represents the strength of correlation. All molecular signatures were included in the network without specifying a cut-off. The protein-metabolite network was generated using (Cytoscape; https://cytoscape.org; v3.10.1)

For post-feature selection analysis, a comprehensive correlation analysis unveiled a rich network of positive associations among the extracted features (Fig. 5a). Within this network, GABA and D-maltose emerged as key players with the highest number of connections, exhibiting synergistic interactions with (inosine-5’-monophosphate dehydrogenase 2; P12268) and the protein group peptidyl-prolyl cis-trans isomerase A-like 4, which includes the following proteins: P12268, A0A075B767, P0DN37, P0DN26, F5H284, A0A0B4J2A2, and Q9Y536. Notably, urea and glycerol 3-phosphate displayed independent positive correlations with the same protein group, peptidyl-prolyl cis-trans isomerase A-like 4. Spermine, on the other hand, adopted a targeted approach, demonstrating direct positive interactions with individual proteins (tropomyosin alpha-4 chain; P67936) and (large ribosomal subunit protein uL2; P62917). Notably, AMP engaged in a distinct network of positive associations with a separate subset of proteins, including calmodulin-1 (P0DP23) and the Rab GDP dissociation inhibitor protein group (P50395 and P31150). Beyond the identified positive associations, a nuanced picture of negative interactions emerged. Notably, L-glutamine, L-pyroglutamic acid, and hypoxanthine exhibited negative correlations with the same profile of protein members, including inosine-5’-monophosphate dehydrogenase 2 (P12268) and the protein group peptidyl-prolyl cis-trans isomerase A-like 4. Additionally, spermine and cytosine showed strong negative associations with structural maintenance of chromosomes proteins (Q9UQE7 and O95347), respectively. The potential biological implications of the protein-metabolite interactions identified by DIABLO are summarized in Supplementary Table S4.

Delving deeper into the interplay between the identified proteomics and metabolomics signatures, a network analysis was conducted (Fig. 5b). As shown in Fig. 5b, a crucial group of hub features, spanning both proteome and metabolome, that serve as key orchestrators within the network were identified. From block one, spermine and L-ornithine were identified as prominent hubs, while block two contributed a distinct set of hub proteins, including inosine-5’-monophosphate dehydrogenase 2 (P12268) and the peptidyl-prolyl cis-trans isomerase A-like 4 protein group.

### Joint pathway analysis of the Toxicoproteo-metabolomic data

To elucidate the biological drivers of TZD-mediated cardiotoxic effects and unveil the interconnected pathways governing these alterations at the metabolomic and proteomic levels, a comprehensive joint pathway analysis was conducted.

**Fig. 6:**
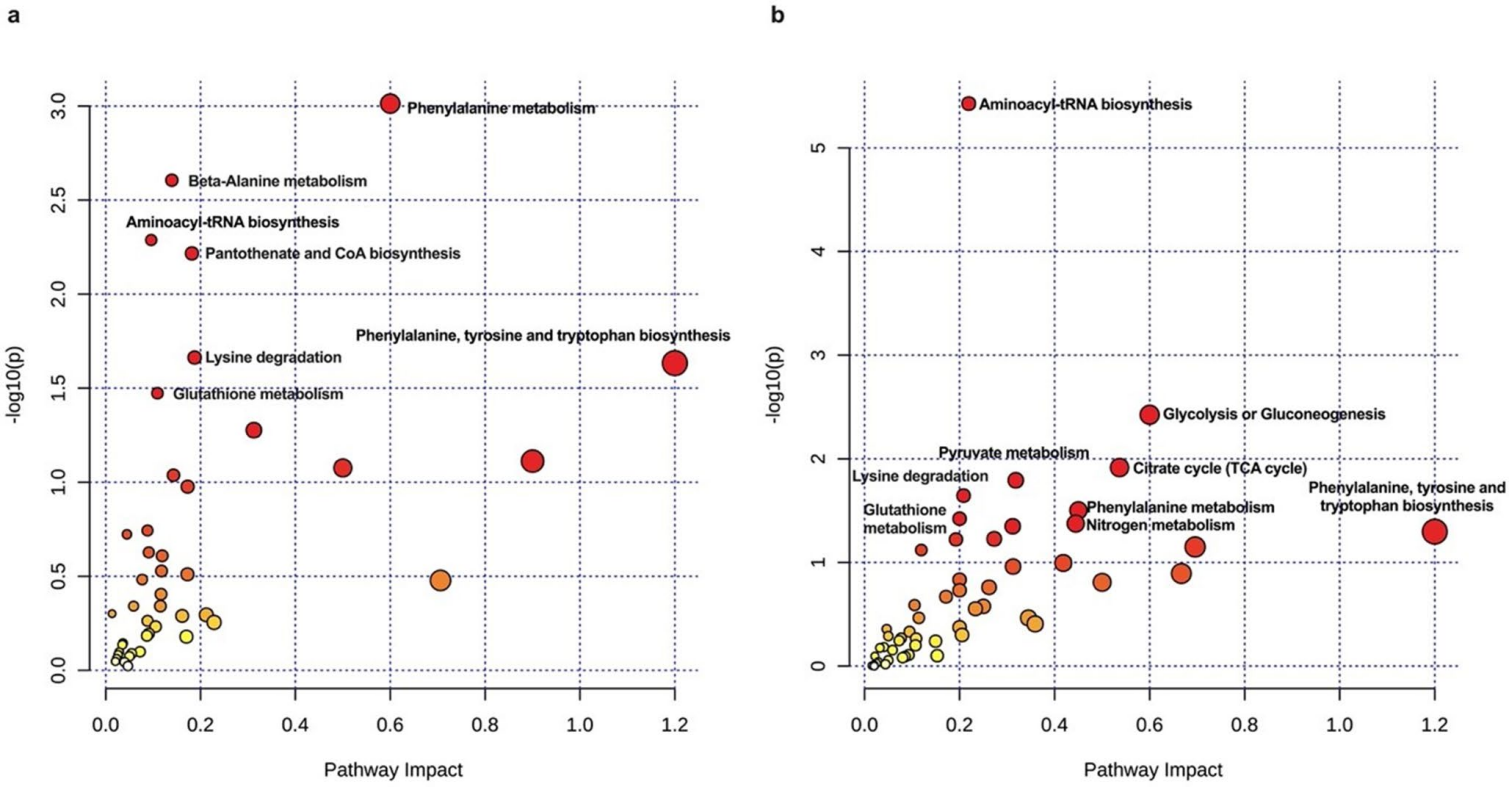
Joint pathway analysis of the proteo-metabolomic data. Dot plots in (**a** and **b**) illustrated the metabolic pathway enrichment in a joint analysis of significantly differentially expressed metabolites and proteins of PGZ and ROSI, respectively. The size and colour of each circle in (**a**) and (**b**) reflect the pathway impact value and the *p*-value, respectively. The figure was generated using MetaboAnalyst v6.0 (https://www.metaboanalyst.ca)

PGZ: Pioglitazone; ROSI: Rosiglitazone.

Analysis of PGZ-treated proteo-metabolomic datasets (Fig. 6a) revealed a pronounced enrichment in pathways related to amino acid metabolism. Notably, pathways associated with phenylalanine metabolism, phenylalanine, tyrosine and tryptophan biosynthesis, glutathione metabolism, beta-alanine metabolism, and lysine degradation exhibited significant enrichment. Additionally, pathways involved in aminoacyl-tRNA biosynthesis and pantothenate and CoA biosynthesis were identified as significantly perturbed in response to PGZ.

Complementary pathway analysis of the ROSI datasets (Fig. 6b) revealed a multifaceted metabolic rewiring encompassing several key functional domains. Pathways involved in core energy production, such as the citrate cycle, pyruvate metabolism, glycolysis/gluconeogenesis, and nitrogen metabolism, showed marked alterations. Strikingly, amino acid metabolism was extensively modulated, with enrichments noted in pathways associated with phenylalanine, tyrosine, and tryptophan biosynthesis; phenylalanine metabolism; arginine and proline metabolism; glutathione metabolism; and lysine degradation. Additionally, perturbations in aminoacyl-tRNA biosynthesis were also observed.

## Discussion

While ensuring the effectiveness of medication in chronic disease is paramount, recognising the importance of managing both safety and cost through a holistic approach, as encapsulated by the medication triangle, contributes to optimal and enduring treatment outcomes. Within the context of T2DM management, TZDs serve as a prime example of how the medication triangle plays out in practice. Despite showcasing efficacy in maintaining glycaemic control and offering affordability, TZDs fall short in the medication triangle, primarily due to concerns surrounding their safety profile (Association, 2023). The emergence of clinical evidence demonstrating a link between TZD usage and HF has fundamentally reshaped the risk–benefit profile of these medications, leading to marked restrictions in their clinical use (Administration, 2010, 2012; De Flines & Scheen, 2007). Nevertheless, the exact mechanisms responsible for triggering or aggravating cardiac events in response to TZD usage are still unclear, impeding a holistic understanding of this complex interplay. Motivated by the obscurity surrounding the mechanistic nature of TZD cardiotoxicity, this study introduced a comprehensive multi-omics approach to unravel the hitherto undeciphered pathomechanisms driving this adverse effect.

### Decoding the metabolic remodeling of AC16 cardiomyocytes following TZD exposure

HF is demonstrably characterised by early disruptions in cardiac energy metabolism, preceding discernible structural alterations. Our multi-level molecular profiling corroborates this notion, uncovering distinct patterns of metabolic reprogramming across multiple pathways, culminating in perturbed cardiac energetics. Our analysis revealed a coordinated downregulation of crucial pathways involving oxidative phosphorylation (OXPHOS), the citric acid cycle (TCA), pyruvate metabolism, and fatty acid synthesis in response to TZD treatment. Conversely, increased activity was observed in glycolysis, the pentose phosphate pathway, and amino and purine metabolism. This coordinated pattern strongly suggests a marked switch in AC16 metabolic fate manifested as metabolic shift from fatty acid oxidation towards anaerobic glycolysis, potentially contributing to cardiotoxicity progression.

In the context of TCA and OXPHOS, a marked downregulation in mitochondrial NAD(P)^+^-dependent malic enzyme (m-NAD(P)-ME), a protein with a prominent role in catalysing the oxidative decarboxylation of malate to pyruvate, feeding into the TCA cycle (Hsieh et al., 2019) was noted following PGZ treatment. However, ROSI treatment induced substantial downregulation of fumarate hydratase (FH), a homotetrameric mitochondrial enzyme catalysing the reversible hydration of fumarate to malate within the TCA (Valcarcel-Jimenez & Frezza, 2023). This downregulation led to a marked accumulation of fumarate, mirroring its observed abundance in TZD-treated cells. Another crucial protein perturbed by ROSI treatment is malate dehydrogenase, a key enzyme in the oxidation of pyruvate and TCA and a member of the malate-aspartate shuttle (Ahn et al., 2020). This metabolic pathway functions as a conduit for electrons generated during glycolysis, facilitating their transfer from the cytosol to mitochondria for OXPHOS (Ahn et al., 2020). Malate dehydrogenase catalyses the reversible conversion of malate to oxaloacetate, enabling NADH transfer from the cytoplasm to mitochondria (Ahn et al., 2020). Therefore, given its established role, disruption of malate dehydrogenase by ROSI critically impairs the malate-aspartate shuttle, leading to reduced NADH transfer to mitochondria and compromising OXPHOS. Furthermore, ROSI treatment induced a striking enrichment in the nitrogen metabolism pathway. Notably, the glutamate dehydrogenases (GLUD1), key enzymes converting glutamate to α-ketoglutarate (α-KG), exhibited significant downregulation (Craze et al., 2019). This resulted in an accumulation of glutamate, as confirmed via our analysis, and compromised aerobic energy output, as α-KG serves as a crucial intermediate in the TCA cycle.

Essential for normal cardiac function, long-chain fatty acids serve as the preferred energy source for the heart, enabling efficient ATP production through mitochondrial β-oxidation while simultaneously contributing to the structural integrity and function of cellular membranes by replenishing their lipid composition (Yamamoto & Sano, 2022). Our complementary analysis revealed a compelling downregulation of the fatty acid synthesis pathway, evidenced by the marked decrease in acyl-CoA synthetase long chain family member 1 (ACSL1) expression in both PGZ- and ROSI-treated cells. This significant ACSL1 downregulation, a key enzyme responsible for long-chain fatty acid activation and β-oxidation initiation (Roelands et al., 2019), aligns with the observed reduction in cellular fatty acid levels, particularly palmitic and stearic acids. These findings, especially following ROSI treatment, suggest a potential impairment in fatty acid oxidation, which could underlie the observed changes in cellular energy metabolism. The present data support the observations of Shekar et al., who reported increased degradation of proteins essential for mitochondrial fatty acid metabolism resulting in deficits in fatty acid oxidation in a Sprague Dawley rat model of transverse aortic constriction-induced moderate HF (Shekar et al., 2014).

Beyond directly impacting fatty acid synthesis and β-oxidation, TZDs orchestrate a broader metabolic reprogramming reverberating through amino acid metabolism pathways markedly linked to fatty acid oxidation. This multifaceted effect, revealed by our integrated pathway analysis, manifests as a significant downregulation of lysine degradation in cardiac cells. This combined pathway analysis unveils an accumulation of lysine and its precursor, L-α-aminoadipate, coupled with a significant suppression of dihydrolipoamide dehydrogenase, a critical pyruvate dehydrogenase complex subunit vital for β-oxidation and pyruvate-to-acetyl-CoA conversion feeding the TCA cycle (Duarte et al., 2021). Additionally, consistent with the lysine degradation pathway, TZD treatment associates with depleted carnitine and its precursor, γ-butyrobetaine (observed in ROSI-treated cells). Given carnitine’s central role in transporting long-chain fatty acids into mitochondria for β-oxidation (carnitine shuttle), this reduced carnitine pool provides a novel mechanistic explanation for the observed perturbation in cardiac energetics following TZD administration, as we described in our previous paper (Al Sultan et al., 2024b). While both medications elicited significant effects on branched-chain amino acid metabolism, our study additionally highlights modulations in the aromatic amino acid pool, particularly L-phenylalanine and L-tyrosine. Despite their marginal contribution as energy substrates, these observations echo prior reports linking such alterations to cardiac remodelling (Geng et al., 2020; Karwi & Lopaschuk, 2023). Nevertheless, the ability of these amino acid fingerprint changes to serve as early biomarkers for subclinical cardiac hypertrophy in the context of acute TZD administration remains elusive and necessitates further research.

TZD treatment of AC16 cells triggered a metabolic shift towards anaerobic glycolysis, orchestrated by the upregulation of key glycolytic enzymes and glucose transporters, such as aldolase A and lactate dehydrogenase A noted in TZD-treated cells. Additionally, overexpression of pyruvate dehydrogenase suggested a compensatory mechanism to decrease mitochondrial oxygen consumption and potentially suppress the TCA cycle. These observations collectively indicate TZD-induced cellular hypoxia, which, along with potential cardiac energy deficits, may drive the observed upregulation of purine metabolism in both drug-treated groups (Doigneaux et al., 2020). The altered expression of Inosine-5’-monophosphate dehydrogenase 1, a key enzyme in de novo guanine nucleotide synthesis (Liu et al., 2023), could explain elevated guanine metabolite levels. Furthermore, upregulation of the purine salvage pathway, as indicated by increased inosine and hypoxanthine, suggests a cellular response to mitigate energy deficits by recovering nucleotides from RNA and DNA degradation (Johnson et al., 2019). Finally, modulated expression of adenylate kinase 6, an enzyme involved in maintaining the nuclear adenine nucleotide pool (Deline et al., 2021), further supports the notion of increased cellular demand for nucleotides under hypoxic and glucose-deprived conditions. Notably, the aforementioned observations regarding perturbations in mitochondrial energetics align with our previously reported in vitro cytotoxicity finding of a significant depletion in mitochondrial ATP upon TZD exposure, underscoring the consistency of these observations and highlighting the potential impact of TZDs on cellular energy production (Al Sultan et al., 2024a).

TZD-induced alterations in cardiomyocyte fatty acid synthesis, β-oxidation, and amino acid and purine metabolism, as previously described, further translate to modulations in cellular redox status, highlighting the multifaceted impact of TZD on energy metabolism. Our analysis revealed a disrupted glutathione (GSH) system upon TZD administration, evidenced by a significant decrease in GSH content. This depletion could potentially stem from elevated reactive oxygen species (ROS) generated due to TZD-induced mitochondrial damage. Further proteomic investigation of TZD-treated cells unveiled perturbations in GSH anabolism, contributing to the diminished intracellular GSH pool. Specifically, downregulation of key enzymes was observed: (i) glutathione synthetase, responsible for the rate-limiting step of GSH synthesis (Tan et al., 2023), and (ii) glutathione disulfide (GSSG) reduction-related enzymes, such as glutathione reductase and glucose-6-phosphate dehydrogenase, crucial for recycling oxidised glutathione (GSSG) back to GSH (Tan et al., 2023). These findings collectively suggest a TZD-induced imbalance in the cellular redox state, rendering AC16 cells more susceptible to ROS damage. Further elucidating the role of oxidative stress in TZD-mediated cytotoxicity, we utilised the fluorogenic dye H_2_DCFDA, a broad-spectrum ROS marker, to quantify intracellular ROS levels in AC16 cells upon TZD exposure. Notably, both PGZ and ROSI induced significant ROS elevation at concentration ranges of 10–100 µM and 1–100 µM, respectively, further supporting the implication of oxidative stress in TZD mediated cytotoxicity. Comprehensive descriptions of the experimental procedures and the corresponding figures are provided in the supplemental materials (Sect. 1.3, Figure S4).

Building upon established biomarkers and leveraging the power of data-driven-based analysis, this study successfully identified key molecular signatures associated with TZD-induced cardiotoxicity across diverse omics datasets using the DIABLO model and the *tune.block.splsda* function. Prominent among these were signatures of amino acids such as L-ornithine, L-tyrosine, and glutamine, known HF biomarkers, further solidifying their potential utility in clinical settings (Geng et al., 2020; Karwi & Lopaschuk, 2023). Similarly, proteomic signatures revealed alterations in energy metabolism pathways (OXPHOS, pentose phosphate pathway, fatty acid synthesis) reflected by proteins such as (NADH dehydrogenase [ubiquinone] 1 alpha subcomplex subunit 9, mitochondrial; Q16795), (6-phosphogluconate dehydrogenase, decarboxylating; P52209), and (fatty acid synthase; P49327), respectively. Interestingly, ROSI datasets yielded distinct protein signatures enriched in energy metabolism (e.g. ATP synthase; O75947) but additionally highlighted disruption of protein synthesis machinery, specifically the peptidyl-prolyl cis-trans isomerase A-like 4 protein group (P0DN37, P0DN26, Q9Y536), suggesting potential endoplasmic reticulum stress and impaired protein export. Notably, these DIABLO-derived signatures align well with the observed metabolic shifts in AC16 cells upon TZD treatment. However, for their translation into clinically relevant prognostic tools for TZD cardiotoxicity and early detection of subclinical hypertrophy, rigorous validation and further investigation are warranted.

### Limitations and future research directions

While the present investigation has yielded valuable insights into our understanding of TZD cardiotoxicity, it is prudent to acknowledge several limitations before drawing definitive conclusions. Motivated by the National Research Council’s (NRC) emphasis on minimising animal experimentation, this study embraced the growing trend of utilising in vitro-to-in vivo extrapolation methodologies in mechanistic toxicology research (Krewski et al., 2020). Accordingly, AC16 cells were chosen as a relevant cell model for investigation. Recognising the AC16 model’s prominent position within the field of cardiac research and its inherent advantages in terms of growth rate and cost-efficiency relative to other models, this study employed this cell line for its investigations. Despite the strengths of the AC16 model, certain limitations warrant consideration. Namely, its dependence on glycolysis, fibroblast-like morphology, and potential for dedifferentiation, along with the complexities of maintaining differentiated cultures (Davidson et al., 2005), restricted our investigation to proliferative cells, as we previously described in (Al Sultan et al., 2024b).

The present study utilises a multi-omics framework, integrating toxicoproteomics and toxicometabolomics analyses, to establish novel causal relationships spanning various molecular levels with unprecedented precision. This comprehensive approach offers significant advantages over single-omics analyses in elucidating the complex interplay between molecular alterations and phenotypic manifestations. However, inherent challenges associated with multi-omics studies, such as variations in technology sensitivity across different investigations and the lack of standardised protocols for sample preparation and data acquisition, can hinder the comparability and reproducibility of findings. Addressing these fundamental limitations is crucial to maximising the advancement and fruitful progress within omics research.

In conclusion, this study pioneers the integration of LC–MS-based toxicoproteomics and toxicometabolomics data to unravel the mechanistic underpinnings of TZD-induced cardiotoxicity. The network analysis of proteo-metabolomic data revealed a notable fingerprint of perturbed biochemical pathways, primarily involving energy metabolism. Additionally, the study identified a marked disruption in the GSH system, indicating an imbalanced redox state triggered by TZD administration. These findings collectively illuminate potential therapeutic avenues, paving the way for future research to improve the safety profile of TZD agents.

## Electronic supplementary material

Below is the link to the electronic supplementary material.

Supplementary Material 1

## Data Availability

All mass spectrometry metabolomics data has been uploaded onto the MetaboLights database and can be found under the MTBLS9279 Study Identifier. All mass spectrometry proteomics data has been uploaded onto the PRIDE database and can be found under the PXD048231 Study Identifier.
