# Supplementary material for: Integrative analysis of toxicometabolomics and toxicoproteomics data: new molecular insights into thiazolidinedione-induced cardiotoxicity
Source: Metabolomics. 2024 Dec 4;21(1):1. doi: 10.1007/s11306-024-02201-3 (PMC11621136; doi:10.1007/s11306-024-02201-3)
Supplement: Supplementary file 1 — Supplementary Material 1 [file 11306_2024_2201_MOESM1_ESM.docx]

**Integrative Analysis of Toxicometabolomics and Toxicoproteomics Data: New Molecular Insights into Thiazolidinedione-Induced Cardiotoxicity**

Abdullah Al Sultan^1,2^, Zahra Rattray & Nicholas J. W. Rattray^1,^*

^1^Strathclyde Institute of Pharmacy and Biomedical Sciences, University of Strathclyde, 161 Cathedral Street, Glasgow, UK, G4 0RE

^2^Faculty of Pharmacy, Kuwait University, Safat, 13110, Kuwait

Corresponding Author [*nicholas.rattray@strath.ac.uk](mailto:*nicholas.rattray@strath.ac.uk)

**Supplementary File**

This file is divided into three sections: supplementary data, supplementary figures and supplementary tables.

# Supplementary Data

## **Toxicometabolomics Pipeline Methodology**

### Sample Preparation and Metabolite Extraction

To profile changes in the endogenous metabolites, AC16 cells were seeded at a density of 2×10^6^ cells/well in six-well plates containing 2 mL of medium per well and incubated for 24 h. Following a 24-h incubation period, the cells were washed once with phosphate-buffered saline (PBS) and supplemented with either a new phenol red-free medium alone or exposed to the half-maximal inhibitory concentration (IC_50_) of either PGZ or ROSI. After the 24 h treatment period, the plates were placed on an ice-cold metal plate, and the AC16 cells were washed with 500 μL of ice-cold PBS. Using a pre-chilled plastic cell scraper, the cells were harvested three times with 500 μL of ice-cold methanol/water (50/50, v/v) and aliquoted in microcentrifuge tubes. Subsequently, the microcentrifuge tubes were placed in liquid nitrogen. The samples were then allowed to sit for a few seconds and vortexed for 2 min. The resultant extracts were centrifuged at 12,000 g for 15 min at 4°C. The supernatant was then collected into new microcentrifuge tubes and evaporated using a Thermo Scientific™ Savant™ SpeedVac™ to form dried metabolite extract pellets, while the recovered sediment pellets were retained for total protein quantification using the Bradford assay. The dried metabolite pellets were reconstituted in water/0.1% formic acid at volumes normalised to the relative protein content. Eventually, the reconstituted solutions were transferred to 300µL fixed insert glass vials for LC-MS analysis. Following sample preparation, quality control (QC) and blank samples were prepared. The QC samples were prepared by mixing equal volumes of all the prepared and tested samples. The blank sample, typically used to monitor background contamination or interference acquired through sample preparation, was prepared by pooling methanol/water (50/50, v/v).

### Micro-flow LC–MS-based Metabolomic Data Acquisition

Metabolite extracts of the AC16 cell biomass and corresponding culture media were randomised and subsequently analysed by high-performance liquid chromatography-electrospray ionisation quadrupole orbitrap mass spectrometry (HPLC-ESI-HRMS) using a Thermo Scientific™ Vanquish™ binary LC system coupled to a Thermo Scientific™ Orbitrap Exploris™ 240 mass spectrometer. The LC separations were carried out on an Accucore C18 HPLC column (2.6 μm, 100 mm × 2.1 mm I.D.; Thermo Fisher) thermostatted at 40°C and operated at a flow rate of 400 μl/min. A 5 μl sample injection was used with an elution gradient consisting of water (eluent A) and acetonitrile (eluent B), each containing 0.1% formic acid (**Table 1**).

**Table 1.** HPLC gradient mode of the mobile phase

| **No.** | **Time** | **Flow (ml/min)** | **%B** |
| --- | --- | --- | --- |
| **1** | 0.000 | Run | |
| **2** | 0.000 | 0.400 | 1.0 |
| **3** | 0.500 | 0.400 | 1.0 |
| **4** | 2.000 | 0.400 | 50.0 |
| **5** | 10.500 | 0.400 | 99.0 |
| **6** | 11.000 | 0.400 | 99.0 |
| **7** | 11.500 | 0.400 | 1.0 |
| **8** | 14.900 | 0.400 | 1.0 |
| **9** | 15.000 | 0.400 | 1.0 |
| **10** |  |  | |
| **11** | 15.000 | Stop Run | |

The mass spectrometer (Orbitrap Exploris™ 240) was equipped with a heated electrospray ion source (HESI-II). The HESI-II was operated in positive (+ESI) mode and set as follows: spray voltage 3700 V; sheath gas flow rate: 40 (arbitrary units); auxiliary gas flow rate: 10 (arbitrary units); sweep gas flow rate: 1 (arbitrary units); ion transfer tube temperature: 300°C; and vaporiser temperature: 280°C. The duty cycle consisted of a full MS scan with an MS1 resolution of 60,000 and then 5 subsequent data dependent acquisition scans using 30,000 resoluiton and an RF lens of 70% in an m/z scan range of 70–1050. XCalibur ™ 4.2 software (Thermo Fisher Scientific) was used for data acquisition.

### Data Processing Using Compound Discoverer 3.2

The acquired LC-MS data were processed using Compound Discoverer 3.2 software (Thermo Fisher, San Jose, CA, USA). Briefly, raw data files were aligned with adaptive curve settings with 3 ppm mass tolerance and a 0.3 min retention time shift. Unknown compounds were detected with 3 ppm tolerance, 30% relative intensity tolerance and 20,000 minimum detectable peak intensity and subsequently grouped by mass tolerance and retention time tolerance by 3 ppm mass and 0.2 min, respectively. Subsequently, the normalisation procedure took place, at which peak areas across all the samples were normalised to the total area of the corresponding samples. Features identified in the processed raw data of mass spectral peaks within a 5-ppm mass error were searched against the mzCloud spectral library and ChemSpider™ databases. Databases selected by ChemSpider were the Human Metabolome Database (HMDB), BioCyc, Chemical Entities of Biological Interest (ChEBI), Kyoto Encyclopaedia of Genes and Genomes (KEGG), Taneisa Grier, Toxin, Toxin-Target Database, WikiPathways and xPharm. All data reported align to MSI Level 2 identification. No in-house chemical standards were run alongside the metabolomics samples during the analysis.

Before the data analyses and through Compound Discoverer 3.2 software, the spectral data were filtered by annotation filters (i.e., a full match with the predefined databases). This was followed by data normalisation using the *MSPrep R package* (Hughes et al., 2014), with the normalisation mode applied being median mode. The data were log_2_ transformed, and no imputation was applied since no missing values were reported in the dataset.

1.1.4 Data Integration Analysis for Biomarker discovery using Latent cOmponents (DIABLO) CODE

The following code was used in the generation of the DIABLO models

protein <- as.matrix(ROSI_P_Normalised_names[, -1]) # Assuming the first column is sample names

metabolite <- as.matrix(ROSI_M_Normalised [, -1]) # Assuming the first column is sample names

X <- list(Proteome = protein, Metabolome = metabolite)

classVector <- factor(rep(c("Ctrl", "ROSI"), each = 9))

Y <- classVector

summary(Y)

##########################################################################################

design <- matrix(0.1, ncol = length(X), nrow = length(X),

dimnames = list(names(X), names(X)))

diag(design) <- 0

design

##########################################################################################graphics.off()

library(mixOmics)

diablo.tcgaA <- block.plsda(X, Y, ncomp = 5, design = design)

perf.diablo.tcgaA = perf(diablo.tcgaA, validation = 'Mfold', folds = 5, nrepeat = 50)

plot(perf.diablo.tcgaA, legend.position = "none" )

perf.diablo.tcgaA$choice.ncomp$WeightedVote

ncomp <- perf.diablo.tcgaA$choice.ncomp$WeightedVote["Overall.BER", "mahalanobis.dist"]

##########################################################################################

test.keepX <- list(Proteome = c(5:9, seq(10, 25, 5)),

Metabolome = c(seq(5, 25, 5)))

tune.diablo.tcgaA <- tune.block.splsda(X, Y, ncomp = 4,

test.keepX = test.keepX, design = design,

validation = 'Mfold', folds = 5, nrepeat = 50,

BPPARAM = BiocParallel::SnowParam(workers = 2),

dist = "mahalanobis.dist")

list.keepX <- tune.diablo.tcgaA$choice.keepX

list.keepX

diablo.tcgaA <- block.splsda(X, Y, ncomp = ncomp,

keepX = list.keepX, design = design)

diablo.tcgaA$design

#######################################################################################

plotDiablo(diablo.tcgaA, ncomp = 1)

#######################################################################################

plotIndiv(diablo.tcgaS, ind.names = FALSE, legend = TRUE, size.legend = 8, ellipse = TRUE,

size.subtitle = 10, legend.title = 'Genotype')

#######################################################################################

#######################################################################################

pdf(width=10, height=5)

plotLoadings(diablo.tcgaS, comp = 4, contrib = 'max', method = 'median', size.title = 8, size.name = 1, border = TRUE,)

dev.off()

#######################################################################################

pdf(width=10, height=5)

loadings_plot <- plotLoadings(diablo.tcga, comp = 2, contrib = 'max', method = 'median', size.title = 8, size.name = 1, border = TRUE,)

loadings_plot +

geom_bar(stat = "identity", position = position_dodge(width = 0.3)) +

theme_minimal()

dev.off()

#######################################################################################

pdf(width=9, height=8)

cimDiablo(diablo.tcgaA, color.blocks = c('darkorchid', 'lightgreen'),

margin=c(8,20), legend.position = "right", size.legend = 0.8, trim = FALSE)

dev.off()

######################################################################################

pdf(width=7.5, height=7)

circosPlot(diablo.tcgaS, ncomp = 4, cutoff = 0.6, line = TRUE,

color.blocks = c('darkorchid', 'lightgreen'),

color.cor = c("red","blue"), size.labels = 1, size.variables = 0.6)

dev.off()

######################################################################################

## **Toxicoproteomics Pipeline Methodology**

### Sample Preparation for Proteomic Profiling

AC16 cells were cultured to 70–80% confluence and seeded at 2×10^6^ cells/well in six-well plates (Cat. No. 140675; Thermo Fisher Scientific, Roskilde, Denmark). Following attachment, the medium was replaced with either fresh medium (control) or medium containing TZD agent at the calculated IC_50_ and incubated for 24 h. Cells were scraped from the six-well plates, aliquoted into 1.5 mL Eppendorf microtubes (Eppendorf, Hamburg, Germany) and centrifuged at 3,000 g for 10 min (4 °C). The supernatant was discarded, and cell pellets retained for proteomics profiling using EasyPep Mini MS Sample Prep Kits in accordance with manufacturer's instructions as described in (Varnavides et al, 2022). In addition to the experimental samples, quality control (QC) and blank samples were prepared. The QC sample was prepared by pooling equal volumes of all the experimental samples, while the blank sample consisted of 50 μL of acetonitrile: water (50:50).

### Micro-flow LC–MS-based Proteomics

## **Data Acquisition**

Peptide separation was performed on a binary Thermo Vanquish ultra-high-performance liquid chromatography system where 20 μL of the reconstituted peptide mixture extract was injected onto a Thermo Acclaim C_18_ PepMap 100 column (150mm x 1mm, particle size 3µm) and separated over a 100min method. The column was maintained at 40 °C, while the autosampler temperature was set at 5 °C. For chromatographic separation, a consistent flow rate of 50 µl/min was used where the mobile phase in positive and negative heated electrospray ionisation mode (HESI+/-) was composed of Solvent A (99.9% water with 0.1% formic acid) and solvent B (99.9% acetonitrile with 0.1% formic acid) (**Table 2**). All post columns viper fittings had a 75 μm internal diameter (black colour code).

**Table 2.** HPLC gradient mode of the mobile phase

| **No.** | **Time** | **Flow (ml/min)** | **%B** |
| --- | --- | --- | --- |
| **1** | 0.000 | Run | |
| **2** | 0.000 | 0.050 | 3.0 |
| **3** | 65.000 | 0.050 | 20.0 |
| **4** | 70.000 | 0.050 | 40.0 |
| **5** | 74.000 | 0.050 | 95.0 |
| **6** | 79.000 | 0.050 | 95.0 |
| **7** | 84.000 | 0.050 | 3.0 |
| **8** | 100.000 | 0.050 | 3.0 |
| **9** |  |  | |
| **10** | 100.000 | Stop Run | |

A high-resolution Exploris 240-Orbitrap mass spectrometer (Thermo Fisher Scientific) was used to perform bottom up proteomics analysis. Operating parameters were set as follows: spray voltages of 3400V in HESI +ve mode. The temperature of the ion transfer tube was set at 320°C with a vaporiser temperature of 75°C. Sheath, aux gas and sheath gas flow rates were set at 25, 5 and 0 Arb, respectively. A Top-20 Data-Dependent Acquisition (DDA) was performed using the following parameter: survey scan range was 275-1500 m/z with MS1 resolution of 120,000, RF Lens of 70% and an intensity threshold of 1.0e^4^. Subsequent MS/MS scans were collected with a resolution of 15,000, isolation window of 1.2 m/z, and with a normalised HCD Collision Energy of 30%. A data dependent cycle time of 3 seconds between master scans was also employed. High-purity nitrogen was used as nebulising and as the collision gas for higher energy collisional dissociation.

**Data Processing**

Using Proteome Discoverer (PD) v3.0 software (Thermo Fisher, San Jose, CA, USA), the MS raw data were used to search the UniProtKB Human Reference Proteome database (v22.07.13; 79,740 entries), including common contaminants (247 entries), using the SEQUEST and Percolator algorithms involved in modified label-free quantification (LFQ) standard processing and consensus workflows. The precursor mass tolerance was set to 10 ppm and 0.02 Da for fragment mass tolerance. Full tryptic digestion was selected, with up to two missed cleavages allowed. The minimum and maximum peptide lengths were set at 6 and 144 amino acids, respectively. Cysteine carbamidomethylation was set as a static modification, and oxidation of methionine (Met), Met loss and N-terminal acetylation were set as dynamic modifications. A maximum of three modifications were allowed per peptide. A concentrated target–decoy approach was applied for the false discovery rate (FDR) calculation, which was set to 1% for highly confident peptide hits.

For protein abundance, the Feature Mapper node was enabled. The raw files were chromatographically aligned with a 10-min retention time shift. The minimum signal-to-noise threshold for feature linking and mapping was set at a value of 5. For precursor peptide abundance, precursor chromatographic intensities were used for precursor quantification results, and the total peptide amount was the selected normalisation mode to correct for experimental bias. Unique and razor peptides were used to quantify the identified proteins.

After processing the raw data of the tryptic peptides using PD, further data filtering (FDR < 1% for both peptides and proteins), normalisation and imputation were conducted via *NormalyzerDE* (Willforss, Chawade, & Levander, 2018) and *NAguideR* (S. Wang et al., 2020) packages, yielding thousands of proteins in both PGZ and ROSI experiments. In both experiments, the data were log_2_ transformed, followed by median normalisation and data imputation using the KNN method.

## **Determination of Reactive Oxygen Species Production**

To examine the ability of TZDs to induce the production of reactive oxygen species (ROS) in the cells, the intracellular level of the ROS was estimated using a fluorescent 2′7′-dichlorodihydrofluorescein diacetate dye (H_2_DCFDA) (Cat. No. D399; Thermo Fisher, Waltham, MA, USA). H_2_DCFDA, a chemically reduced form of fluorescein, is a nonpolar compound converted into a polar and membrane-impermeable derivative of H_2_DCF under the presence of cellular esterases (Wu & Yotnda, 2011). The latter compound is nonfluorescent (Wu & Yotnda, 2011). However, upon oxidation through the intracellular level of ROS, H_2_DCF is oxidised to highly fluorescent 2',7'-dichlorofluorescein (DCF) (Wu & Yotnda, 2011).

Cells were seeded in a dark, clear-bottomed 96-well microplate (50,000 cells/well) and incubated overnight. Following 24 h of incubation, the cells were washed once with PBS and loaded with 5 μM H_2_DCFDA for 30 min at 37 °C in the dark. Subsequently, the dye was removed, and the cells were exposed to various concentrations of PGZ or ROSI (1, 5, 10, 50, and 100 µM) and incubated for 6 h. DCF fluorescence was measured using a FlexStation 3 Multi-Mode Microplate Reader (Molecular Devices, Sunnyvale, CA, USA) at the maximum excitation and emission spectra of 492 and 517 nm, respectively.

# Supplementary Figures


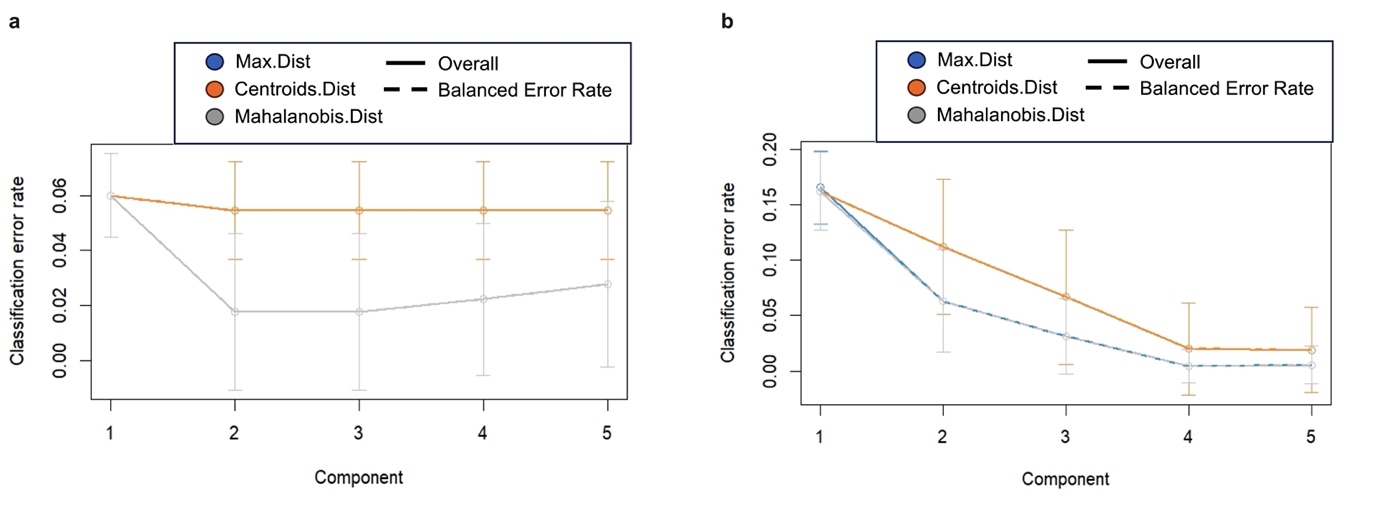


**Figure S1. Evaluation of MB-sPLS-DA model performance via repeated cross-validation.** MB-sPLS-DA classification performance was evaluated using repeated cross-validation (50 × 5-fold) for each component, considering both overall and balanced error rate across different prediction distances (max.dist, centroids.dist, mahalanobis.dist) in the PGZ (a) and ROSI (b) studies.  The bars show the standard deviation across the repeated folds. The plots in (a and b) show that the error rate reaches a minimum of two and four components, respectively.

MB-sPLS-DA: Multiblock sparse partial least squares discriminant analysis; PGZ: Pioglitazone; ROSI: Rosiglitazone; BER: Balanced error rate


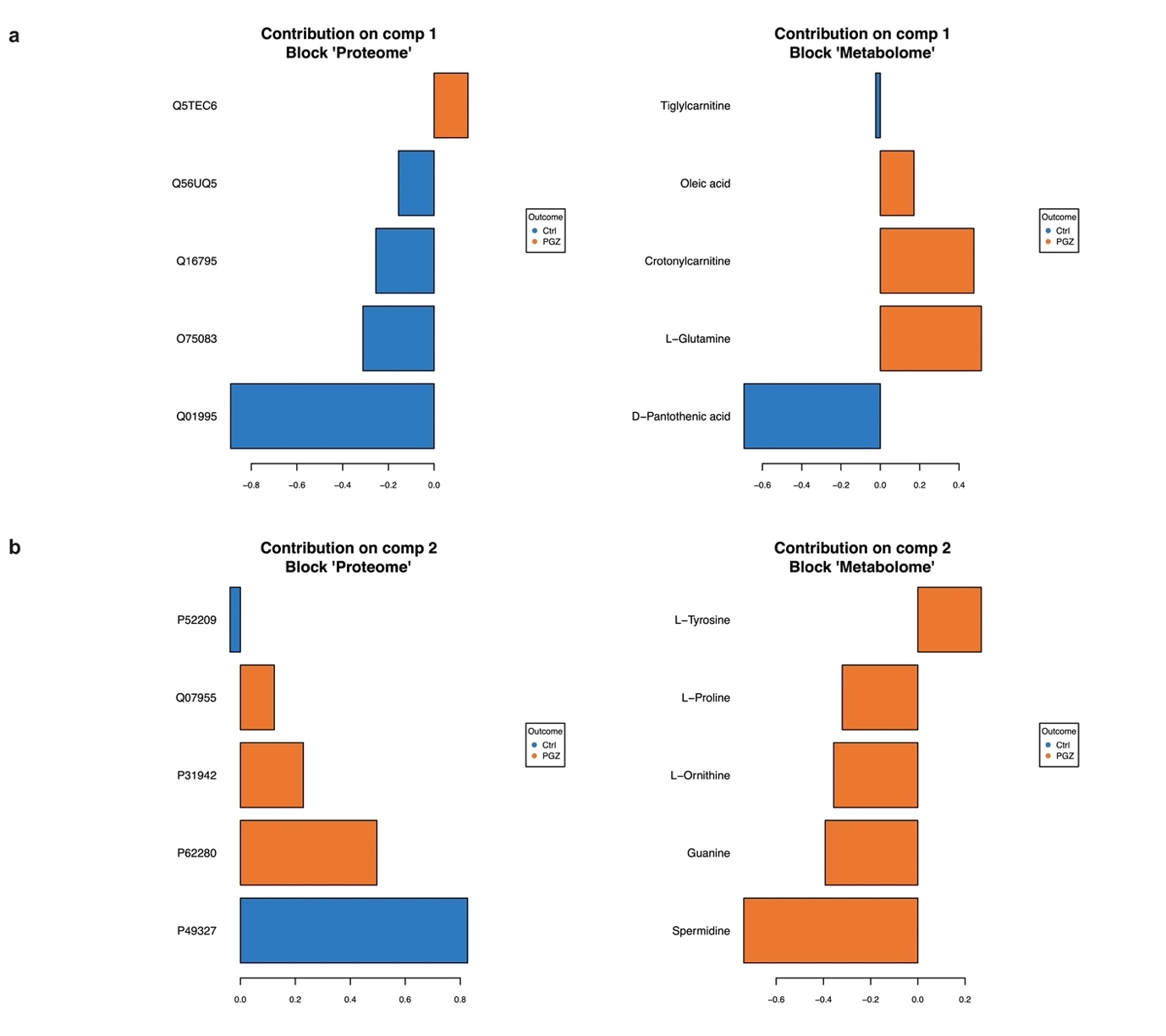


**Figure S2. Loading plot for the molecular signatures selected by MB-sPLS-DA performed in the PGZ study.** The plots (a and b) display the most important variables, ranked by the magnitude of their coefficients from bottom to top. Each variable's colour denotes the class with the highest median expression level.

MB-sPLS-DA: Multiblock sparse partial least squares discriminant analysis; PGZ: Pioglitazone


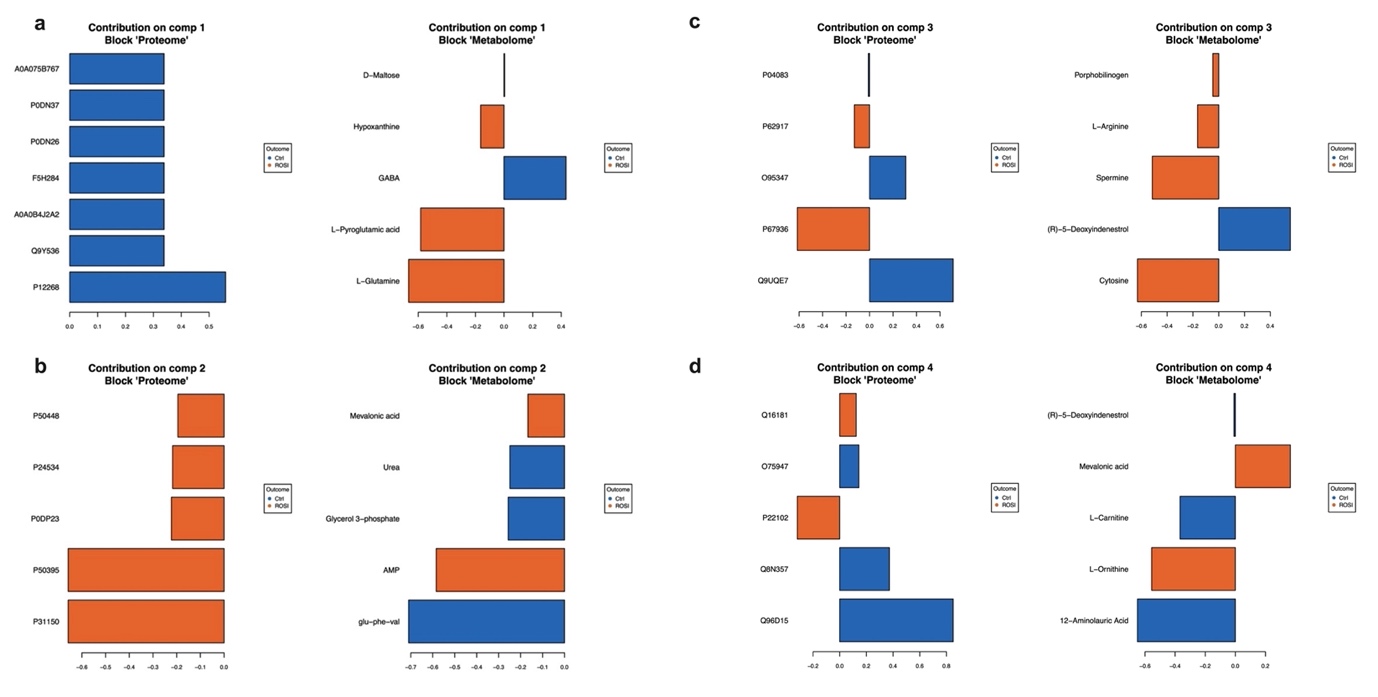


**Figure S3. Loading plot for the molecular signatures selected by MB-sPLS-DA performed in the ROSI study.** The plots (a-d) display the most important variables, ranked by the magnitude of their coefficients from bottom to top. Each variable's colour denotes the class with the highest median expression level.

MB-sPLS-DA: Multiblock sparse partial least squares discriminant analysis; ROSI: Rosiglitazone


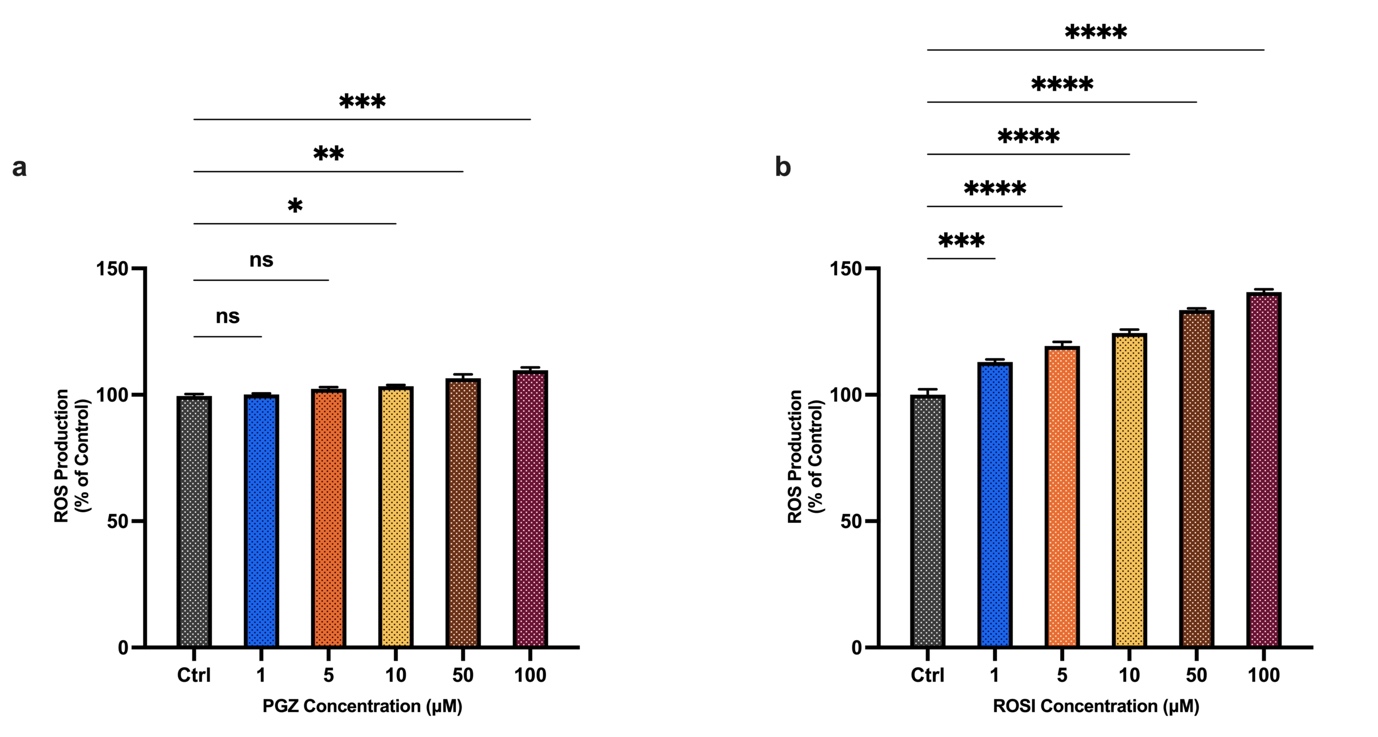


**Figure S4. Effect of TZDs on ROS production.** The AC16 cells were pre-cultured for 24 h in 96-well plates (50,000 cells/well). Afterwards, the cells were loaded with 5 µM H_2_DCFDA and exposed to various concentrations of either PGZ (a) or ROSI (b). The data are from three independent experiments, each performed in triplicate and are expressed as the mean ± SD. Statistical significance was determined using a one-way ANOVA and Dunnett’s multiple comparisons test (control vs. each concentration of TZD).

* *p* < 0.05, ** *p* < 0.01, *** *p* < 0.001 and **** *p* < 0.0001

TZDs: Thiazolidinediones; ROS: Reactive oxygen species; PGZ: Pioglitazone; ROSI: Rosiglitazone; ns: Non-significance

# Supplementary Tables

**Table S1. List of signature proteins identified by the DIABLO model in the PGZ study.**

| **Accession** | **Protein Name** | **Gene Symbol** | ***p*-value** |
| --- | --- | --- | --- |
| Q56UQ5 | TPT1-like protein | *TPT1* | 3E-10 |
| Q16795 | NADH dehydrogenase [ubiquinone] 1 alpha subcomplex subunit 9, mitochondrial | *NDUFA9* | 3E-09 |
| Q5TEC6 | Histone H3-7 | *H3-7* | 8E-08 |
| O75083 | WD repeat-containing protein 1 | *WDR1* | 1E-06 |
| P62280 | Small ribosomal subunit protein uS17 | *RPS11* | 4E-01 |
| Q01995 | Transgelin | *TAGLN* | 5E-07 |
| P49327 | Fatty acid synthase | *FASN* | 4E-01 |
| Q07955 | Serine/arginine-rich splicing factor 1 | *SRSF1* | 2E-01 |
| P31942 | Heterogeneous nuclear ribonucleoprotein H3 | *HNRNPH3* | 3E-02 |
| P52209 | 6-phosphogluconate dehydrogenase, decarboxylating | *PGD* | 3E-01 |

**Table S2. List of signature proteins identified by the DIABLO model in the ROSI study.**

| **Accession** | **Protein Name** | **Gene Symbol** | ***p*-value** |
| --- | --- | --- | --- |
| P67936 | Tropomyosin alpha-4 chain | *TPM4* | 4E-01 |
| Q9UQE7 | Structural maintenance of chromosomes protein 3 | *SMC3* | 4E-01 |
| O95347 | Structural maintenance of chromosomes protein 2 | *SMC2* | 5E-01 |
| Q8N357 | Solute carrier family 35 member F6 | *SLC35F6* | 3E-01 |
| Q16181 | Septin-7 | *SEPTIN7* | 3E-01 |
| P62917 | Large ribosomal subunit protein uL2 | *RPL8* | 4E-01 |
| Q96D15 | Reticulocalbin-3 | *RCN3* | 3E-01 |
| A0A075B767 | Peptidyl-prolyl cis-trans isomerase A-like 4H | *PPIAL4H* | 5E-05 |
| P0DN37 | Peptidyl-prolyl cis-trans isomerase A-like 4G | *PPIAL4G* | 5E-05 |
| P0DN26 | Peptidyl-prolyl cis-trans isomerase A-like 4F | *PPIAL4F* | 5E-05 |
| F5H284 | Peptidyl-prolyl cis-trans isomerase A-like 4D | *PPIAL4D* | 5E-05 |
| A0A0B4J2A2 | Peptidyl-prolyl cis-trans isomerase A-like 4C | *PPIAL4C* | 5E-05 |
| Q9Y536 | Peptidyl-prolyl cis-trans isomerase A-like 4A | *PPIAL4A* | 5E-05 |
| P12268 | Inosine-5'-monophosphate dehydrogenase 2 | *IMPDH2* | 9E-05 |
| P50395 | Rab GDP dissociation inhibitor beta | *GDI2* | 7E-02 |
| P31150 | Rab GDP dissociation inhibitor alpha | *GDI1* | 7E-02 |
| P22102 | Trifunctional purine biosynthetic protein adenosine-3 | *GART* | 5E-01 |
| P24534 | Elongation factor 1-beta | *EEF1B2* | 4E-01 |
| P0DP23 | Calmodulin-1 | *CALM1* | 5E-01 |
| O75947 | ATP synthase subunit d, mitochondrial | *ATP5PD* | 7E-02 |
| P04083 | Annexin A1 | *ANXA1* | 2E-01 |
| P50448 | Factor XIIa inhibitor | *SERPING1* | 1E-03 |

**Table S3. Potential biological implications of protein-metabolite interactions identified by DIABLO framework in the PGZ dataset**

| **Protein** | **Metabolite** | **Type of Interaction** | **Biological Perspective** | **Reference(s)** |
| --- | --- | --- | --- | --- |
| Heterogeneous nuclear ribonucleoprotein H3 | L-tyrosine | Positive | Plays a role in RNA processing and transport. L-tyrosine supports RNA processing and transport by contributing to protein stability, phosphorylation-dependent regulation, and RNA stabilization, essential for efficient RNA splicing and nuclear export. | (Gu et al, 2020) |
| 6-Phosphogluconate dehydrogenase | L-ornithine / guanine | Positive | This interaction is primarily associated with the pentose phosphate pathway and nitrogen metabolism. L-ornithine and guanine may regulate the enzyme's activity in NADPH generation, which is essential for biosynthetic processes, redox homeostasis, and nucleotide synthesis. | (Pan et al, 2024) |
| TPT1-like protein | D-pantothenic acid | Positive | This interaction contributes to cellular energy metabolism and stress adaptation. D-pantothenic acid, a precursor for Coenzyme A, is crucial for numerous metabolic pathways, including fatty acid oxidation, the TCA cycle, and amino acid metabolism. | (Czumaj et al, 2020) |
| NADH dehydrogenase (mitochondrial) | D-pantothenic acid | Positive | Essential in mitochondrial ATP production, this interaction enhances electron transport chain function and CoA-dependent metabolic pathways, as NADH dehydrogenase is part of the electron transport chain and D-pantothenic acid supports CoA synthesis, a cofactor in ATP synthesis. | (Czumaj et al, 2020) |
| Histone H3-7 | D-pantothenic acid | Negative | This interaction may impact chromatin structure and gene expression by influencing histone acetylation through D-pantothenic acid-mediated acetyl-CoA synthesis. | (Simithy et al, 2017) |
| Fatty acid synthase | Spermidine | Negative | Spermidine, a polyamine involved in various cellular processes, might modulate fatty acid synthesis by targeting fatty acid synthase. This potential regulatory mechanism may serve to balance cellular lipid and polyamine metabolism. | (Zhou et al, 2022) |

**Table S4. Potential biological implications of protein-metabolite interactions identified by DIABLO framework in the ROSI dataset**

| **Protein** | **Metabolite** | **Type of Interaction** | **Biological Perspective** | **Reference(s)** |
| --- | --- | --- | --- | --- |
| Inosine-5'-monophosphate dehydrogenase 2 | GABA | Positive | GABA might interact with IMPDH to regulate purine synthesis during metabolic stress, contributing to cellular homeostasis. | (Su et al, 2024) |
| Peptidyl-prolyl cis-trans isomerase A-like | D-maltose | Positive | D-maltose may influence peptidyl-prolyl isomerase’s role in protein folding and stability, particularly under energy-demand conditions. | (Jewett et al, 2009) |
| Inosine-5'-monophosphate dehydrogenase 2 | D-maltose | Positive | D-maltose might interact with IMPDH to regulate energy output, linking carbohydrate metabolism with purine biosynthesis. | (Wu et al, 2023) |
| Peptidyl-prolyl cis-trans isomerase A-like | Urea | Positive | Elevated urea levels may suggest cellular stress, where the isomerase aids in protein stabilization under increased nitrogen waste. | (Perrucci et al, 2015) |
| Tropomyosin alpha-4 chain | Spermine | Positive | Spermine likely stabilizes tropomyosin, supporting cytoskeletal organization and cellular integrity. | (Sagar et al, 2021) |
| Rab GDP dissociation inhibitor protein group | AMP | Positive | AMP may modulate Rab GTPase activity through its effects on Rab GDP dissociation inhibitors, thereby impacting vesicular trafficking. | (Shinde and Maddika, 2018) |
| Inosine-5'-monophosphate dehydrogenase 2 | L-glutamine/ L-pyroglutamate | Negative | Elevated L-glutamine levels may downregulate IMPDH activity, potentially affecting purine nucleotide biosynthesis. | (Gonzalez Herrera et al, 2018) |
| Inosine-5'-monophosphate dehydrogenase 2 | Hypoxanthine | Negative | Hypoxanthine may downregulate IMPDH activity, acting as a negative feedback regulator of de novo purine biosynthesis. | (Gonzalez Herrera et al, 2018) |
| Spermine, AMP, and ornithine | | | Spermine, AMP, and ornithine are interconnected biomolecules that play crucial roles in cellular growth, energy metabolism, and protein synthesis. Ornithine, a key intermediate in the urea cycle, is a precursor for putrescine, which is further metabolized into spermidine and spermine. These polyamines are essential for DNA and RNA stabilization, as well as various cellular processes. AMP, as a nucleotide, is involved in energy transfer and signaling pathways. It provides the energy required for polyamine synthesis and other cellular processes, including protein synthesis and DNA replication. | (Sagar et al, 2021) |

**References**

Czumaj, A., Szrok-Jurga, S., Hebanowska, A., Turyn, J., Swierczynski, J., Sledzinski, T., & Stelmanska, E. (2020) The pathophysiological role of CoA. *International Journal of Molecular Sciences*, 21(23), 9057.

Gu, J., Chen, Z., Chen, X., & Wang, Z. (2020) Heterogeneous nuclear ribonucleoprotein (hnRNPL) in cancer. *Clinica Chimica Acta*, 507, 286-294.

Herrera, K. N. G., Zaganjor, E., Ishikawa, Y., Spinelli, J. B., Yoon, H., Lin, J. R., ... & Haigis, M. C. (2018) Small-molecule screen identifies de novo nucleotide synthesis as a vulnerability of cells lacking SIRT3. *Cell reports*, 22(8), 1945-1955.

Jewett, M. C., Miller, M. L., Chen, Y., & Swartz, J. R. (2009) Continued protein synthesis at low [ATP] and [GTP] enables cell adaptation during energy limitation. *Journal of bacteriology*, 191(3), 1083-1091.

Ou, W. B., Luo, W., Park, Y. D., & Zhou, H. M. (2001) Chaperone‐like activity of peptidyl‐prolyl cis‐trans isomerase during creatine kinase refolding. *Protein Science*, 10(11), 2346-2353.

Pan, Y., Li, Y., Chhetri, J. K., Liu, P., Li, B., Liu, Z., ... & Ma, L. (2024) Dysregulation of acyl carnitines, pentose phosphate pathway and arginine and ornithine metabolism are associated with decline in intrinsic capacity in Chinese older adults. *Aging Clinical and Experimental Research*, 36(1), 36.

Perrucci, G. L., Gowran, A., Zanobini, M., Capogrossi, M. C., Pompilio, G., & Nigro, P. (2015) Peptidyl-prolyl isomerases: a full cast of critical actors in cardiovascular diseases. *Cardiovascular research*, 106(3), 353-364.

Sagar, N. A., Tarafdar, S., Agarwal, S., Tarafdar, A., & Sharma, S. (2021) Polyamines: functions, metabolism, and role in human disease management. *Medical Sciences*, 9(2), 44.

Shinde, S. R., & Maddika, S. (2018) Post translational modifications of Rab GTPases. *Small GTPases*, 9(1-2), 49-56.

Simithy, J., Sidoli, S., Yuan, Z. F., Coradin, M., Bhanu, N. V., Marchione, D. M., ... & Garcia, B. A. (2017) Characterization of histone acylations links chromatin modifications with metabolism. *Nature communications*, 8(1), 1141.

Su, R., Chang, L., Zhou, T., Meng, F., & Zhang, D. (2024) Effects of GABA on Oxidative Stress and Metabolism in High-Glucose Cultured Mongolian Sheep Kidney Cells. *International Journal of Molecular Sciences*, 25(18), 10033.

Varnavides, G., Madern, M., Anrather, D., Hartl, N., Reiter, W. & Hartl, M. (2022) In search of a universal method: a comparative survey of bottom-up proteomics sample preparation methods. *Journal of Proteome Research*, 21(10), 2397-2411.

Wu, D. & Yotnda, P. (2011) Production and detection of reactive oxygen species (ROS) in cancers. *JoVE (Journal of Visualized Experiments)*(57), e3357.

Wu, Z., Bezwada, D., Cai, F., Harris, R. C., Ko, B., Sondhi, V., ... & DeBerardinis, R. J. (2024) Electron transport chain inhibition increases cellular dependence on purine transport and salvage. *Cell Metabolism*.

Xuan, M., Gu, X., Li, J., Huang, D., Xue, C., & He, Y. (2023) Polyamines: their significance for maintaining health and contributing to diseases. *Cell Communication and Signaling*, 21(1), 348.

Zhou, J., Pang, J., Tripathi, M., Ho, J. P., Widjaja, A. A., Shekeran, S. G., ... & Yen, P. M. (2022) Spermidine-mediated hypusination of translation factor EIF5A improves mitochondrial fatty acid oxidation and prevents non-alcoholic steatohepatitis progression. *Nature Communications*, 13(1), 5202.
